# Medication in AN: A Multidisciplinary Overview of Meta-Analyses and Systematic Reviews

**DOI:** 10.3390/jcm8020278

**Published:** 2019-02-25

**Authors:** Corinne Blanchet, Sébastien Guillaume, Flora Bat-Pitault, Marie-Emilie Carles, Julia Clarke, Vincent Dodin, Philibert Duriez, Priscille Gerardin, Mouna Hanachi-Guidoum, Sylvain Iceta, Juliane Leger, Bérénice Segrestin, Chantal Stheneur, Nathalie Godart

**Affiliations:** 1 Maison de Solenn-Maison des Adolescents, Cochin Hospital, Assistance Publique-Hôpitaux de Paris, 75014 Paris, France; corinne.blanchet@aphp.fr (C.B.); marie-emilie.carles@aphp.fr (M.-E.C.); 2 CESP, INSERM 1178, Paris-Descartes University, USPC, 75014 Paris, France; 3 French Federation Anorexia Bulimia (FFAB), 75014 Paris, France; s-guillaume@chu-montpellier.fr (S.G.); flora.bat@ap-hm.fr (F.B.-P.); julia.clarke@aphp.fr (J.C.); dodin.vincent@ghicl.net (V.D.); phduriez@gmail.com (P.D.); priscille.gerardin@chu-rouen.fr (P.G.); mouna.hanachi@aphp.fr (M.H.-G.); sylvain.iceta@chu-lyon.fr (S.I.); berenice.segrestin@chu-lyon.fr (B.S.); chantal.stheneur@fsef.net (C.S.); 4 Department of Psychiatric Emergency & Acute Care, Lapeyronie Hospital, CHRU Montpellier, 34090 Montpellier, France; 5 INSERM U1061, University of Montpellier, 34090 Montpellier, France; 6 Child and Adolescent Psychopathology Unit, Salvator Hospital, Public Assistance-Marseille Hospitals, 13009 Marseille, France; 7 Institut de la Timone, CNRS, Aix-Marseille University, 13005 Marseille, France; 8 Child and Adolescent Psychiatry Department, Robert Debré Hospital, Assistance Publique-Hôpitaux de Paris, 75019 Paris, France; 9 INSERM U894, Institute of Psychiatry and Neuroscience of Paris (IPNP), 75013 Paris, France; 10 Clinique Médico-Psychologique, Neurosciences Hôpital Saint Vincent de Paul, 59000 Lille, France; 11 Faculté de Médecine et de Maïeutique de Lille, 59800 Lille, France; 12 Sainte-Anne Hospital (CMME), Paris Descartes University, 75014 Paris, France; 13 Pôle universitaire de psychiatrie de l’enfant et de l’adolescent CH du Rouvray-CHU de Rouen, 76300 Rouen, France; 14 CRFDP, UFR des Sciences de l’Homme et de la Société, Rouen University, 76130 Mont-Saint-Aignan, France; 15 Clinical Nutrition Unit, Raymond Poincaré University Hospital, Assistance Publique-Hôpitaux de Paris, 92380 Garches, France; 16 CESP, INSERM, UMR 1018, University Paris-Sud, UVSQ, University Paris-Saclay, 94800 Villejuif, France; 17 Referral Center for Eating Disorder, Hospices Civils de Lyon, 69677 Bron, France; 18 Equipe PSYR2, INSERM U1028, CNRS UMR5292, Université Lyon 1, 69002 Lyon, France; 19 Pediatric Endocrinology Diabetology Department, Reference Centre for Endocrine Growth and Development Diseases, Robert Debré University Hospital, Assistance Publique-Hôpitaux de Paris, 75019 Paris, France; juliane.leger@aphp.fr; 20 Paris Diderot University, Sorbonne Paris Cité, F-75019 Paris, France; 21 Institut National de la Santé et de la Recherche Médicale (INSERM), UMR 1141, DHU Protect, F-75019 Paris, France; 22 INSERM 1060, Laboratoire CARMEN, Centre de Recherche en Nutrition Humaine Rhône-Alpes, Claude Bernard University, Lyon 1, Pierre Bénite, 69310 Lyon, France; 23 Centre Médical et Pédagogique, Fondation Santé des Etudiants de France, 91480 Varennes Jarcy, France; 24 Faculté de Médecine, Université de Montréal, Quebec, QC H3C 3J7, Canada; 25 Adolescent and Young Adult mental health department, Fondation Santé des Etudiants de France, 75014 Paris, France; 26 UFR Simone Veil-Santé, 78690 Saint-Quentin en Yvelines, France

**Keywords:** anorexia nervosa, drug-treatment, pharmacotherapy, medication, nutrition, comorbidity, complication

## Abstract

Drugs are widely prescribed for anorexia nervosa in the nutritional, somatic, and psychiatric fields. There is no systematic overview in the literature, which simultaneously covers all these types of medication. The main aims of this paper are (1) to offer clinicians an overview of the evidence-based data in the literature concerning the medication (psychotropic drugs and medication for somatic and nutritional complications) in the field of anorexia nervosa since the 1960s, (2) to draw practical conclusions for everyday practise and future research. Searches were performed on three online databases, namely MEDLINE, Epistemonikos and Web of Science. Papers published between September 2011 and January 2019 were considered. Evidence-based data were identified from meta-analyses, if there were none, from systematic reviews, and otherwise from trials (randomized or if not open-label studies). Evidence-based results are scarce. No psychotropic medication has proved efficacious in terms of weight gain, and there is only weak data suggesting it can alleviate certain psychiatric symptoms. Concerning nutritional and somatic conditions, while there is no specific, approved medication, it seems essential not to neglect the interest of innovative therapeutic strategies to treat multi-organic comorbidities. In the final section we discuss how to use these medications in the overall approach to the treatment of anorexia nervosa.

## 1. Introduction

Anorexia nervosa (AN) is a severe condition with high morbidity and mortality rates resulting from both somatic and psychiatric aspects of the disorder [1]. International guidelines recommend treatment based on a multidisciplinary approach, including nutritional, somatic, psychiatric, and social aspects [2,3,4,5]. This global treatment is mainly based on nutritional and psychotherapeutic approaches associated with the treatment of medical and socio-familial complications. In the National Institute for Health and Care Excellence (NICE) guidelines [4] it is clearly mentioned that medications cannot be seen as the sole treatment for anorexia nervosa (Number 1.3.24 see Table 1). 

As stated by Aigner et al. [6] the treatment objectives in AN include weight gain, prevention of weight loss, a change in eating behaviours, reduction of associated psychopathologies (e.g., preoccupations with body image, depression, anxiety, obsessive compulsive symptoms) and the treatment of associated medical conditions (e.g., disturbances of the gonadal axis, infertility, osteoporosis). Medication for its part is used to treat AN symptoms including eating disorder symptoms (eating attitudes, refusal to gain weight, preoccupation about shape and weight, obsessions about food…) and psychiatric symptoms (depression, anxiety, obsessions, and compulsions.). In addition, AN patients frequently have severe malnutrition and medical complications that can be treated by medication. Indeed, while the return to a normal weight by re-feeding associated with multidisciplinary care is crucial and enables the correction of many somatic functional disorders, it seems essential not to neglect the usefulness of somatic medication in limiting the short and long-term physical complications, optimizing quality of life and promoting a favourable outcome in all fields affected by AN (somatic, psychiatric and social). 

In 2011 the World Federation of Societies of Biological Psychiatry (WFSBP) guidelines for the pharmacological treatment of eating disorders [6] concluded that the majority of the drugs to treat AN are used off-label [6]. These drugs are widely prescribed, but not in compliance with international guidelines [7,8], and can therefore be potentially unsafe for patients with AN. 

For everyday practice, clinicians need improved recommendations about what medications can be used, for what purpose and in which conditions. Current guidelines give some indications for the use of different medications, but they lack precision and do not define who can be treated, when, and with which medication (as seen in Table 1, NICE recommendations for medication in AN). When clinicians try to find information in the literature, it is not easy, as there are numerous publications on the subject. For example, since the WFSBP guidelines were established for the pharmacological treatment of eating disorders, there have been around 300 reports on the topic of medication and AN, and no review covering all drugs used in the nutritional, somatic, and psychiatric fields.

The main aim of this work is to offer clinicians a multidisciplinary overview of the evidence-based published literature concerning drug treatments in the field of AN. This overview is based on the conclusions of meta-analyses, failing that on systematic reviews, and otherwise on trials (randomized or if not open-label studies). Unlike other reviews, this one is not restricted to drugs used to treat AN symptoms or psychiatric symptoms, but also includes the treatment of the medical conditions and the nutritional aspects associated with AN. 

## 2. Methods 

A systematic literature search was performed on the topic of medication in AN in order to perform a systematic overview, according to the following method.

### 2.1. Data Sources and Search Strategies

Searches were performed on online databases, namely MEDLINE, Epistemonikos and Web of Science. Inclusion and exclusion criteria are described in Table 2. 

Only meta-analyses and systematic reviews with a detailed methodology were retained; narrative or qualitative reviews were excluded, as they provide elements only on some studies, and can be biased by a lack of information. We excluded studies that investigated mixed eating disorder groups, as their conclusions cannot be extended to AN.

The searches were complemented by a manual search: reference lists of articles were manually investigated to identify reviews or meta-analyses potentially relevant for inclusion that were not detected by electronic search. 

The search strategies were conducted in two phases by two of the investigators (C.B., N.G.).

First, we defined a search in order to identify all reviews and meta-analyses published on the topic of drug treatments in AN with the following terms and algorithm, which, among the algorithms tested with different key words and combinations, proved to be the one that retrieved the largest corpus of papers: “anorexia nervosa and pharmacotherapy or drug treatment or medication or nutrition or enteral nutrition or weight restoration”.

**Figure 1:**
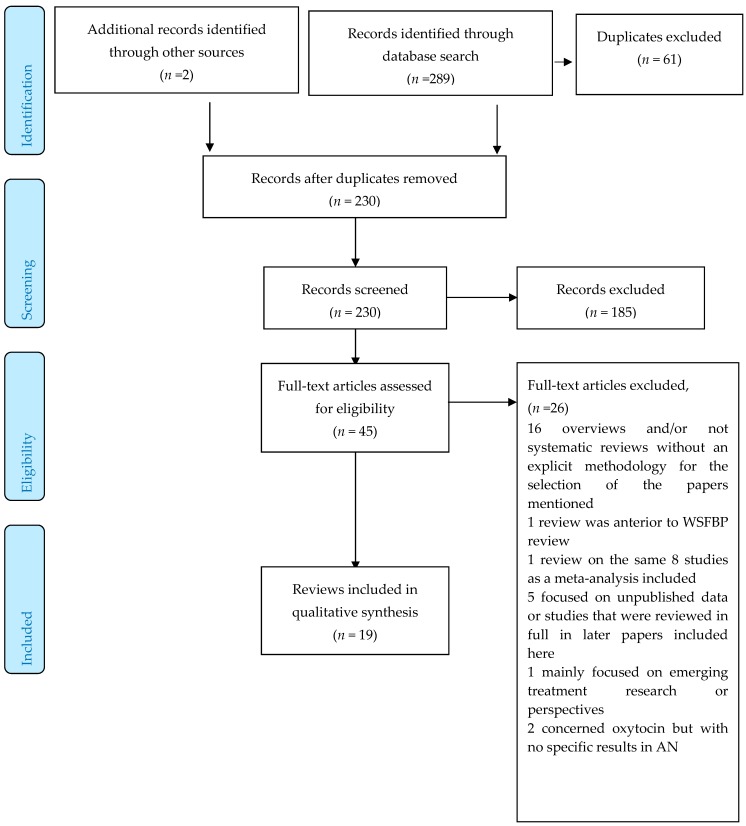
Preferred Reporting Items for Systematic Reviews and Meta-Analyses (PRISMA) Flow Diagram [36].

The search on the three databases previously mentioned selected 289 papers, 45 of which were reviews or meta-analyses on the topic (see Preferred Reporting Items for Systematic Reviews and Meta-Analyses (PRISMA) flow-chart Figure 1). Two further papers were identified by manual research. 

Among the 45 reviews or meta-analyses, 26 were excluded for the following reasons: 16 were overviews and/or not systematic reviews with no explicit methodology for the selection of the papers mentioned [9,10,11,12,13,14,15,16,17,18,19,20,21,22,23,24], one review [25] predated the World Federation of Societies of Biological Psychiatry review, one [26] reviewed the same eight studies as a meta-analysis included [27], five focused on unpublished data, or studies that were reviewed in later papers included here [28,29,30,31,32], one mainly focused on emerging treatment research or perspectives [33], two concerned oxytocin but with no specific results in AN [34,35]. 

The 19 selected papers (15 systematic reviews and four meta-analyses) are presented in Table 3.

Secondly, the overview was completed by original research (RCTs, open studies as mentioned previously) published after the last review or meta-analysis reviewed. In addition, for nutritional and somatic aspects, as there was no meta-analysis nor systematic review on some of the subjects (vitamin D and calcium, micronutrients supplementation and functional digestive disorders), we reviewed published papers (RCTs, and if not open studies as mentioned previously), and retrospective or case studies if no other information existed on the topic of AN. For functional digestive disorders, as there were no papers about their treatment in the AN literature, reviews including this topic (in general and not in AN) were selected. 

### 2.2. Study selection and Quality Assessment

From the French Anorexia and Bulimia Federation (http://www.anorexieboulimie-afdas.fr/), we recruited a group of eating disorder specialists participating on a voluntary basis, including nutritionists, an endocrinologist, an adolescent paediatrician, psychiatrists, and child and adolescent psychiatrists (see authors of the paper).

Eligible papers were screened in the literature search by their title and abstract by two reviewers working together (C.B. and N.G.). The papers were selected by agreement on the basis of the inclusion exclusion criteria (Table 2). Then, the screened papers (meta-analyses or systematic reviews) were read in full and again selected or not, according to inclusion and exclusion criteria. If there was any uncertainty regarding the eligibility of a paper, it was referred to the rest of team for further discussion.

Disagreements between reviewers were resolved by consensus; they mainly concerned the suitability of studies for the purpose of the review.

## 3. Results

### 3.1. Papers Selected

The 19 selected systematic reviews are presented in Table 3. 

We gave priority here to meta-analyses and systematic reviews rather than to study reports, according to the following rules. For each topic, meta-analysis results are presented first when available. Then, if the meta-analyses only focused on a particular period or a specific question, or if there was no meta-analysis on the topic, the results of the more recent systematic reviews on the topic based on RCTs were described, and completed by previous ones if they provided more information. If reviews on the topic were not available, existing trials on the topic are reported, RCTs are reported in priority if available, if not open trials are reported. Study reports are only cited if they were not referenced in the cited meta-analyses or reviews and were published after the reviews (we did not take into account papers excluded from the reviews for methodological reasons).

We do not report on case reports, or retrospective chart studies, as they are based on the local practice of the teams involved, and are more a reflection of their practice than an evaluation of treatments. In case of numerous reviews or meta-analyses on a topic, results are completed by earlier reviews if they provide complementary information. When samples only involved adolescents, this is mentioned, and any specifics concerning the adolescents are also mentioned.

### 3.2. Results: Psychotropic Medications

#### 3.2.1. Methodological Issues

The reviews and meta-analyses selected in this overview reviewed from six [51] to 66 studies [45] (see Table 3). This wide range results from numerous factors (aim of the review; types of study considered: ranging from only RCTs to all types of study; period; methodology for selection of studies: ranging from all published studies to a selection based on quality guidelines; type and number of databases used). For example, Brockmeyer et al. used the Cochrane Handbook and the National Institute of Heath Criteria for a quality assessment of controlled intervention studies [51] between October 2011 and the end of 2016 and selected only three good quality RCTs on psychotropic medication. Conversely, Franck et al. [45] were exhaustive and included all studies (except case reports) published in the past 50 years. Miniati et al. [48] underlined the impossibility of applying quality criteria to select studies in their review. In addition, samples were very different in terms of age (see Table 3), some focused on adolescents, others on adults, and some on both. 

Psychotropic medications have been used since the 1960s to treat AN. For the studies included in the reviews, Miniati et al [48] underlined the paucity of reports, including the small number of RCTs, the small numbers of subjects included in the studies (one to maximum 93 in this review), the heterogeneity of sample composition, treatments, and treatment settings, and the small number of males to enable comparisons based on gender, which are all aspects that impact the conclusions. 

The variable settings (inpatient/outpatient) had an impact on patient characteristics; for example in the studies, these authors observed that the age of inpatients was significantly younger than that of outpatients [48]. In addition, in cases of positive results in favour of a medication, the results had never been replicated. The conclusions of these studies were based on small samples and the fact that they derived no significant results could be partly explained by a lack of power; the results were often extrapolated across patient groups of different ages, illness durations, and severity. Another important limitation of these studies is their duration [48]. Most of the studies were based on short term follow-up in an illness requiring long-term management [48]. The majority of the studies were conducted on inpatient samples, whereas the majority of patients are treated on an ambulatory basis [48]. Not all symptom dimensions of AN, nor all the comorbidities, nor the impact of the medication on these elements were systematically reported.

A small number of studies specially focused on children and adolescents. The majority of the studies were conducted in the acute phase of AN and a minority in the maintenance phase [40]. Below we provide information in terms of dose, duration of treatment, and sample size, for comparative studies only.

#### 3.2.2. Antidepressants (AD)

The rationale for treating AN with antidepressants was initially that AN and depression had clinical and biological similarities, including comorbidity and symptom overlap with anxiety disorders, obsessive compulsive disorders and depression, and a hypothetical dysfunction in the serotonergic and noradrenergic systems in the pathophysiology of AN. The earliest studies mostly concerned tricyclics and monoamine oxydase inhibitors and the more recent mostly concerned selective serotonin reuptake inhibitors (SSRI) [6,37,45,48]. We found one small meta-analysis [41] pooling two studies on tricyclics [54,55], two on selective serotonin reuptake inhibitors [56,57], and five reviews concerning each different class of antidepressant separately [6,37,40,45,48]. No other recent study was found.

##### Meta-analyses

The meta-analyses (see Table 3 for details) concerned all antidepressant RCTs versus placebo evaluating the impact on either weight restoration or maintenance, with various doses, durations and evaluation criteria. It pooled four RCTs. One was on clomipramine (50 mg, duration not mentioned, there were eight placebo subjects and there were eight AD subjects, evaluation criterion: weight gain in kg) [54], one was on amitriptyline (160 mg max, for 32–45 days, there were 25 placebo subjects, and 24 AD subjects, evaluation criterion: weight gain per day) [55]. Two were on fluoxetine [56,57] (respectively 60 mg and 20 to 60mg, for 7 weeks and 1 year, 16 placebo and 44 subjects, 15 AD and 49 subjects, evaluation criteria, respectively: ideal body weight and body mass index) involving 96 patients in the antidepressant groups and 94 patients in the placebo groups. No impact of antidepressants on weight gain or maintenance in AN was found [41]; because of the small sample sizes no meta regression or subgroup analyses were conducted. 

##### Tricyclics

On the basis of an analysis of three RCTs versus placebo [45,48], there is no clear evidence for the general use of tricyclics among patients with AN in terms of weight gain and depressive symptoms, according to two studies on amitryptiline (160 mg max and 2.8 ± 0.3mg/kg, respectively for 32–45 days and five weeks, placebo 25 subjects and 11, AD 24 and 11 subjects, evaluation criteria: weight gain per day and weight plus psychological outcome) [58] and one with clomipramine (50 mg, 76 days clomipramine and 72 days placebo, placebo eight and AD eight subjects, evaluation criterion: weight gain in kg) [54,59]. These drugs only showed some impact on hunger, appetite, and energy intake at the beginning of treatment, but with no effect on weight, as mentioned previously. Although clomipramine tended in one study [54] to be associated with lower weight gain compared to the placebo, this was hypothesised to be linked to more physical activity. An open trial comparing paroxetine to clomipramine (respectively 18.4 ± 4.7 mg and 75.3 ± 7.6 mg, duration 39 ± 26 and 58 ± 30, 39 and AD 57 subjects, evaluation criteria: BMI, duration to weight gain) found no difference in terms of weight gain in an adolescent sample, but duration to obtain weight gain was significantly shorter for paroxetine (72 versus 97 days) [45,60]. In addition, because of the lethal risk with overdose in suicide attempts and the potential for fatal arrhythmia at low body weight, particularly among young subjects, this type of medication is no longer studied and is not recommended today for AN [6,37,40,45,48].

##### Selective Serotonin Reuptake Inhibitors (SSRIs)

There is today no clear evidence supporting the use of SSRIs in AN [48]. 

The three RCTs [56,57,61] on fluoxetine versus placebo provided controversial results (respectively 60 mg for the first and for the others 20 to 60mg, for seven weeks, one year, and 52 weeks, placebo 16, 44, and 16 subjects AD 15, 49, and 19 subjects). Most RCTs with placebo (2/3) reported negative results concerning the effect on eating psychopathology and weight gain [56] and on weight maintenance on a large sample at 12 months [45,48,57]. The only one that had positive results for the maintenance of weight at 12 months and for anxiety [61] had only 13 completers for the treatment and no associated psychological treatment [48]. Some authors hypothesised that fluoxetine inefficacy could be linked to malnutrition, and in particular, to a lack of dietary tryptophan. Tryptophan supplementation in addition to fluoxetine was evaluated in a double-blind controlled trial versus placebo (fluoxetine 20 to 60 mg, for six months, AD and placebo 11 subjects, AD with nutritional supplementation 15 subjects). Barbarich, et al. [62], did not show benefits from this supplementation on weight gain, anxiety or obsessive compulsive symptoms [45]. Three RCTs compared fluoxetine to nortriptyline, amineptine [63,64] (respectively for fluoxetine 60 mg, versus nortriptyline 75 mg, amineptine 300 mg for 16 weeks, 22 subjects and 13 subjects) or clomipramine / amisulpride [65] (respectively for fluoxetine 28 mg, clomipramine 58 mg/ amisulpride 50 mg for 12 weeks, 35 subjects), and failed to demonstrate any difference [40].

The effect of citalopram was only investigated in three open trials [66,67,68]. The authors of the third study with a control group (citalopram 20 mg, for 12 weeks, 19 subjects compared to 20 patients on a waiting list) found no improvement in terms of weight, but an improvement in depression, obsessive-compulsive symptoms, impulsiveness and trait-anger [48], and body dissatisfaction [45]. 

A small open-label study that compared sertraline with the placebo [69] reported that sertraline improved depressive symptoms, perceptions of ineffectiveness, a lack of interoceptive awareness, and perfectionism, but not weight gain [45]

##### Other Antidepressants 

Monoamine oxydase inhibitors were evaluated in an open study among six patients for six weeks [70]. Mood and anxiety improved [45] but these drugs are no longer used, as a result of both their inefficacy on weight and their unfavourable side effect profile [48]. Venlaxine has only been studied in an open trial [71], in comparison with fluoxetine and in association with cognitive behavioural therapy, with no differences in weight or behaviour outcomes [45]. Mirtazapine and duloxetine used in AN have only been described in case reports on adults [48]. Concerning adolescents, mirtazapine was not found to be superior to other medications nor to no medication in AN [45]. 

In conclusion, antidepressants have no impact on weight gain [45,48], and their impact on eating symptoms or psychopathology is not clear. Tricyclics and monoamine oxydase inhibitors have adverse side effect profiles and should no longer be used in AN [45,48].

##### Antipsychotics

The rationale for treating AN with antipsychotics was initially linked to the hypothesis that obsessions regarding weight and body shape in AN (abnormal beliefs that are ego-syntonic and characterised by an acute lack of insight that persists even when the affected person’s health status is endangered) could be viewed as delusional ideas and consequently could result from dopamine receptor hypersensitivity in AN [45]. Other arguments for their use in AN were based on the effect of these drugs on reward system regulation, their potential efficacy in controlling problematic frequency of physical activity (usually called hyperactivity in AN) and the weight gain side effects observed in other disorders [48]. Another recent argument is that second generation drugs also act on the salience network, known to be impaired in eating disorders, and that they could thus act by enhancing the reactivity of the anterior cingulate cortex and the salience network in the response to the reward value of food in AN. These drugs are mainly dopamine D2 and serotonin 5HT2A antagonists [71]. Finally, more recently, Frieling et al. [72] cited by Miniati et al. [48], have postulated the existence of an altered expression of the dopaminergic genes among patients exhibiting psychomotor hyperactivity. These drugs are dopamine D2 receptor antagonists with severe adverse effects. For second-generation drugs, the arguments also include positive effects on depression and anxiety symptoms arising from eating disorders [48]. First-generation antipsychotics (typical antipsychotics) and second-generation (atypical) antipsychotics have all been studied.

Four meta-analyses [27,38,41,43] and six reviews concerned each class of antipsychotic separately [6,37,40,45,48,51]. The reviews are considered for first-generation drugs, as they are mainly not included in meta-analyses, and also for second-generation drugs after 2012 (the most recent data in the meta-analyses). Since the end of the last review [48] five study reports have been published about antipsychotics in AN, two retrospective chart reviews on adults and adolescents with AN respectively [45] not reviewed here, one open-label study among adolescents [73] and one among adults [74] not reviewed here, and two RCTs [75,76]. Details concerning antipsychotic studies are presented in Table 4. 

##### Meta-Analyses

The meta-analyses compared antipsychotics, mainly second-generation, to placebo in six to eight studies, but never exactly the same drugs, always with a majority of studies on olanzapine. 2/4 pooled first and second-generation antipsychotics [27,41], the two others only included second-generation drugs [38,43]. They concerned respectively:

- Eight RCTs (four on olanzapine [81,82], one on quetiapine [88], one on risperidone [86], one on pimoside [77] and one on sulpiride [78] versus placebo) with 102 subjects in the antipsychotic group and 114 in the comparator group (six placebo groups and one treatment-as-usual), one study [78] included one sample of adolescents, the last one [86] examined the effects on BMI, weight, glucose levels, depressive and anxiety symptoms, dropout rates from any cause, adverse effects, akathisia and drowsiness/sedation [27].

- Seven RCTs including five against placebo, four on olanzapine [81,82,83,84], one on risperidone [86], and two against chlorpromazine [80] or clomipramine or fluoxetine [65] with 72 subjects in the second-generation antipsychotic group and 75 in the comparator group, exploring weight gain and drive for thinness, body dissatisfaction, overall eating disorder symptoms and anxiety [38,43]. 

- Six RCTs (five on olanzapine [81,82,84,85] and one on sulpiride [78]), including one sample of adolescents [85], one with 73 subjects in the antipsychotic group and 72 in the placebo group, exploring weight post-treatment [41]. 

- Seven RCTs (four on olanzapine [81,82,83,84], two on quetiapine [87,88], and one on risperidone [86] with 91 subjects in the second-generation antipsychotic group and 99 in the placebo group, exploring BMI change, overall changes in anorectic symptoms, and number of dropouts [43]. 

No impact of antipsychotics was found in comparison to placebo on weight gain, whether antipsychotics were pooled or considered individually (evaluated in 4/4 meta-analyses), nor on eating disorder symptoms (evaluated in 3/4 meta-analyses [38]), nor was it found for akathisia [27], dropout [27], or glucose levels [27]. A recent paper by Attia et al. [76] reported an RCT on olanzapine increased to the maximum of 10 mg/day in four weeks compared to placebo. This study was conducted on a large sample of 152 adults AN (83 completers) with a long illness duration (mean age for placebo group 28 ± 10.9 and for olanzapine group 30.0 ± 11.0 years old and illness duration respectively 10.5 ± 9.5 and 12.6 ± 11.7). It showed a modest therapeutic effect of four months of olanzapine, compared with placebo on BMI (with an increase difference of 0.165 BMI points more per month). This study found no difference between groups in terms of clinical global impressions, obsessionality, anxiety and depressive symptoms, nor eating disorders symptoms (except an increase in shape concerns that was observed in the olanzapine group). 

Drowsiness/sedation occurred significantly more often with antipsychotics than with placebo/usual care in the pooled analyses, and especially for olanzapine in an individual analysis [27]. Attia et al [76] found no significant differences in the frequency of the abnormal blood test to assess metabolic abnormalities between the olanzapine and placebo groups. 

For the effect on anxiety and depressive symptoms, there was no apparent efficacy of antipsychotics according to the largest meta-analysis on the topic (pooling four studies) [27]. A recent small RCT study lasting three months among 30 adult outpatients with AN mentioned a superiority of Olanzapine (2.5 mg the first month and 5 mg the two following months) combined with cognitive behavioural therapy (CBT) versus placebo combined with CBT in improving obsessiveness-compulsivity, depression, anxiety and especially hostility (but not weight gain or specific aspects related to the AN eating pathology) without showing results [75].

##### Typical Antipsychotics other than those Explored in the Meta-Analyses

Haloperidol and chlorpromazine have generally not been evaluated in RCTs, and evidence levels for their efficacy and safety are poor [48]. Haloperidol as an adjunct to psychotherapy was found to be associated with weight over six months in one open study [45,48,79]. In an RCT, there was no difference in terms of weight gain between chlorpromazine and olanzapine [80] but olanzapine was superior for the reduction of anorexic ruminations [37].

##### Other Second-Generation Antipsychotics than those Explored in the Meta-Analyses

Amisulpride was studied in one study [45]. It was found to be superior to fluoxetine and clomipramine in a single blind RCT for weight gain (not for weight phobia, body image disturbance or amenorrhea) [65]. 

Aripiprazole was considered only in a case series of adults and young people [48].

In conclusion, according to the four meta-analyses considered here, antipsychotics had no impact on weight gain compared to placebo, and their impact on eating symptoms and psychopathology is not clear. Olanzapine had a modest effect on weight gain in one RCT including adults with a long duration of AN [76]. Typical antipsychotics have an adverse side effect profile and should not be used in AN, except perhaps haloperidol in severe AN [45]. Atypical antipsychotics could alleviate some symptoms, such anorexic ruminations, anxiety or depressive symptoms, but levels of evidence are low, and based only some small studies [75] and not supported by meta-analyses on the topic [27].

#### 3.2.3. Lithium 

The rationale for using lithium among patients with AN is mainly related to the fact that it induces weight gain in other disorders [37,48].

We found four reviews concerning lithium [6,37,48] all mentioning one RCT [89], but we found no other recent study.

This small RCT, involving lithium among adults (dose not mentioned, 16 patients and controls, evaluation criterion: weight gain at weeks three and four) showed a significant difference in terms of weight for the lithium-treated group, but not for other psychological dimensions [89]. However, the use of lithium is not recommended in AN, even for patients with severe and resistant forms. Sodium and fluid depletion are frequent in AN and could reduce lithium clearance, which could lead to lithium poisoning [45,48], as renal complications are frequent in AN [90].

#### 3.2.4. Appetite Enhancers 

##### Antihistamines

Four reviews [6,37,45,48] mentioned antihistamines but we did not find any more recent studies.

Cyproheptadine (CYP), a serotonin and histamine antagonist reputed to produce weight gain among children with asthma, was tested in two RCTs [37,48]. The first RCT in four arms compared Placebo and CPY both with and without cognitive behavioural therapy (12 mg to 32 mg maximum, duration not mentioned, 81 subjects in four groups: CYP and behavioural therapy or placebo, placebo and behavioural therapy, placebo; evaluation criterion: weight gain) [91]. The second compared placebo, CYP and amitriptyline (CPY 32 mg maximum, amitriptyline 160 mg maximum, duration: to 5% of target weight, 72 subjects in three groups, evaluation criterion: duration to 5% target weight) [92]. There was no clinically significant effect on weight gain with CYP in the first RCT. In the second, cyproheptadine marginally decreased the length of time to reach the weight gain objective for restricting AN patients, but it significantly impaired treatment efficacy for the binging/purging anorectic patients, compared to amitriptyline and placebo [45,48]. Thus antihistamines remain non-indicated in AN and should be avoided. No further study was found.

##### Opiates

One review [37] mentioned a study about cannabinoids and we did not find any more recent studies.

The opioid peptide system has been implicated in appetite and feeding regulation, linked to the hedonic value of food in both animals and humans. It has been hypothesised that both anorexia nervosa and bulimia nervosa could be opioid-mediated addictions [37]. In line with these hypotheses opiates and opiate antagonists have been used in order to stimulate eating in AN or to deactivate the suspected auto-addictive properties of food restriction. No study has been conducted in AN exclusively to test this hypothesis. Only one review by Aigner et al. [6] mentions one RCT that was conducted in a mixed sample of 19 AN binging/purging subtype and bulimia nervosa patients using naltrexone, 100 mg for 6 weeks [93]. Bingeing and purging behaviours decreased among both AN and bulimia nervosa patients. Naltrexone has no indication in AN. 

##### Cannabinoids

Given the well-known impact of cannabis on appetite, cannabinoids have also been used and tested in AN. [45,51].

Two reviews mentioned cannabinoids, but we did not find any more recent studies [45,51]. 

Two RCTs evaluated cannabinoids. An earlier four-week double-blind crossover study [45] compared delta-9-terahydrocannabiol (delta-9-THC) to diazepam (delta-9-THC, 7.5 to 30 mg versus diazepam 1 to 15 mg, for four weeks, 11 subjects, evaluation criteria: weight gain, daily calorie intake), and showed no benefit of delta-9-THC [94]. In addition, three patients experienced severe dysphoric reactions under 9-Tetrahydrocannabinol administration. The second four-week crossover RCT (5 mg, 4 weeks, 24 subjects, evaluation criteria: weight gain, daily calorie intake), [95] compared low doses of dronabinol (a synthetic form of delta-9- tetrahydrocannabinol) to a placebo and observed a small significant gain of 0.73 kg for dronabinol. It had no impact on the total duration of physical activity but increased the average intensity of this physical activity [51]. 

In conclusion, cannabinoids have no proof of their efficacy nor of their safety in AN, they need further evaluation in AN, and should not be used in routine care practice. 

##### Ghrelin

One review [45] mentioned ghrelin agonists, but we did not find any more recent studies. 

In a small open trial, infusions of ghrelin over 14 days were delivered to five individuals with AN, and improved gastrointestinal discomfort and improved nutritional intake and weight gain were rapidly observed [96]. 

In conclusion, however, ghrelin has not proved its efficacy nor its safety in AN, and needs more evaluation in that setting. 

#### 3.2.5. Other Medications

##### Benzodiazepines 

Only one review mentioned benzodiazepines [45], and we did not find any more recent studies.

Benzodiazepines are anxiolytic agonists of the gamma-aminobutyric acid (GABA) receptors. Although they are widely used in anorexia nervosa [7] studies that systematically investigated benzodiazepines in AN are scarce, and fairly recent [45]. An RCT on alprazolam in an AN inpatient setting comparing 75 mg of alprazolam prior to a laboratory test meal to placebo, did not find this drug beneficial in the treatment of AN [45]: alprazolam does not improve calorie intake and increases fatigue without reducing anxiety [97].

##### Clonidine

Clonidine is an alpha two adrenergic agonist used to treat hypertension. It was mentioned in only two reviews [6,48] reporting one placebo-controlled crossover study on four patients on 500–700 micrograms/day [98] and we did not find any more recent studies. No beneficial effect in AN was observed, but it was associated with hemodynamic side effects such as hypotension. 

In conclusion, clonidine has not proved its efficacy in AN and should not be used in routine care practice. 

##### N-Methyl-D-Aspartate Agonists and Antagonists

D-cycloserine is an N-methyl-D-aspartate (NMDA) receptor agonist known to facilitate extinction learning, a promising treatment for anxiety disorders. Only one review mentioned glutamatergic drugs [45], and they were reported in two RCTs. We did not find any more recent studies.

In one RCT [99], which used D-cycloserine versus placebo prior to meal exposure therapy, it was found that the D-cycloserin group was linked to a greater weight gain after four exposure sessions and at one-month follow-up [45].

The NMDA receptor antagonist amantadine was also used in a case series of 22 patients [100]. Amantadine administered 45 min before the main meal improved neuro-autonomic symptoms during the meal. Patients were able to eat all types of foods and their BMI increased over three months [45].

In conclusion, these drugs have no proof of their efficacy nor of their safety in AN and they need more evaluation in AN. They should not be used in routine care practice. 

##### Oxytocin

Oxytocin is a neuropeptide hormone synthesised in the hypothalamus. It plays a role in pair bonding and in the regulation of broader social interactions, emotional reactivity and feeding behaviours. Some authors suggest that oxytocin could be a useful adjunct for the treatment for AN [51], but no review has published results. We found one recent RCT. The usefulness of oxytocin as a therapeutic agent in AN was tested in only one RCT in the course of hospital-based nutritional rehabilitation, comparing 16 AN patients under oxytocin 36 UI (intra-nasal) per day for four to six weeks and 17 patients receiving placebo. The weight gain was similar in the two groups, while eating concerns and cognitive rigidity lowered after oxytocin treatment [101].

### 3.3. Results: Somatic and Nutritional Treatments

Recent progress in understanding and progress in care provision for eating disorders has led to an overall consensus at the beginning of the 21st century on somatic and nutritional treatments in multidisciplinary approaches, and on evidence that weight restoration is a key aspect for the correction of many somatic functional disorders. Nevertheless, somatic medications or nutritional approaches for AN patients are more often used off-label, with no detailed guidelines for severe AN inpatient populations, nor for specific organic complications (osteoporosis, growth or puberty failure) treated by various highly specialized physicians.

Our literature review found various narrative reviews concerning somatic aspects of AN, but only a few recent systematic reviews concerning multi-organic somatic medications. In fact, most systematic reviews are concerned with the effects of weight gain or pharmacological treatments (hormone replacement, biphosphonates, teriparatide, and vitamin K) on bone mineral density and secondary osteoporosis [39,44,49,50] or they concerned nutritional therapeutic modalities and their impact on weight changes [42,46], or the efficacy of nasogastric enteral nutrition and adverse effects [47,52,53]. Most of the studies involved small samples, with heterogeneity within and among studies concerning evaluations, biomarkers and age range, with heterogeneous adolescent and adult populations, and various durations, often with an insufficient follow-up. There are very few studies only on child/adolescent populations, and more than 95% of the data mainly concerned female and Caucasian AN patients. 

#### 3.3.1. Nutritional Support and Refeeding 

While weight gain and progressive weight restoration is an important first step in treating patients with AN, and is essential for medical stabilization before starting specific psychiatric care, it is clear that there is a lack of empirical evidence concerning initial refeeding strategies, and that heterogeneous medical practices are observed in everyday practice. 

##### Approaches to Refeeding

One systematic review [42], including seven studies assessed and summarized nutritional treatments provided for 403 adolescent AN inpatients. Initial energy intake, regardless of refeeding protocols, ranged from 1000 kcal to >1900 kcal/day with a maximum energy intake during hospitalization ranging from 2000 to 4350 kcal/day. The maximum energy intake achieved was greater in the groups with additional enteral feeding (three comparative studies out of seven). The level of evidence for these results was not sufficient to propose any consensus on the most effective refeeding protocols, but it supported the need for future research on this topic. 

One systematic review [46], including 27 studies and 2635 patients examined approaches to refeeding among adolescent and adult AN patients in various treatment settings, and 96% were observational/prospective or retrospective and conducted in hospital. This review focused on refeeding protocol analyses, patient clinical characteristics and somato-psychic outcomes. Thirteen studies described a meal-based approach to refeeding (calorie intake divided into meals and snacks), ten studies approached the topic with various combinations of nasogastric feeding and oral intake, one combined total parenteral nutrition and oral intake, three involved altered nutrient content (differing from current dietary recommendations). The main results of this systematic review concluded that the classic refeeding approach (starting the calorie level between 1000 and 1200 kcal/j) among mildly and moderately malnourished patients is too “conservative”, and could be associated with lower weight gain and longer hospitalization. Higher calorie intake (calorie starting level between 1500 and 2400 kcal/j) with a meal-based approach, or a combination of nasogastric feeding and oral intake could be safe and well tolerated with appropriate monitoring. In the absence of sufficient evidence, a lower calorie approach in refeeding remains the rule for severely malnourished inpatients. Parenteral refeeding was associated with multiple adverse effects and is not recommended. The authors of the review suggested more research to evaluate the impact of different refeeding approaches on the duration of a hospital stay and long-term outcomes.

##### Enteral Feeding (EF)

Enteral feeding is indicated if under-nutrition is severe (BMI < 13) and/or associated with metabolic disorders, and/or if there is prolonged weight stagnation, despite adequate nutritional and psychiatric management [3]. EF is considered safe and well tolerated, and effectively enhances calorie intake and the rate of weight gain among patients with AN [102]. Enteral nutrition should always be performed using a small nasogastric tube. Although a few studies reported using percutaneous endoscopic gastrostomy [103], this route should not be used in the nutritional management of anorexia nervosa, because it can aggravate the distortion of body shape perception among patients. An isocaloric and isoprotidic solute should be used continuously (1 mL = 1 kcal) in the first days in case of severe undernutrition, in order to avoid post-stimulatory hypoglycaemia [104]; nocturnal refeeding can also be performed. Caloric progression should be cautious in the first days, beginning with 10–15 kcal/kg/d, and increasing slowly up to 30 to 40 kcal/kg/d at one week, in order to prevent refeeding syndrome [105], but some recent data also reported the potential risk of "underfeeding syndrome", supporting the interest of more aggressive refeeding therapies [106]. EF should be maintained only as needed, to ensure that patients retain normal eating behaviours. Progressive oral feeding should always be encouraged and accompanied by an experienced dietician [107]. 

One recent systematic review [53] including 10 studies, confirmed that EF is a safe therapeutic tool that is well tolerated for the management of AN patients, with an average weight gain > 1kg/week and enhanced calorie intake and weight gain in the four studies comparing EF to oral-only refeeding. Long-term effects associated with nasogastric enteral refeeding are only reported in the RCT study [108], with a higher mean body weight at 12 months in the EF group.

One systematic review [47] including 18 studies and 1427 adolescent and adult AN patients (1406 F/21 M), evaluated physiological and psychiatric outcomes and patient adherence to nasogastric feeding (NG). It can be noted that 95% of the studies were conducted in inpatient medical or psychiatric units and only one study concerned ambulatory patients [109]. Continuous NG was reported for 50% of the patients, and various tube refeeding methods (combined, overnight, bolus, not reported) for the others. Mean duration for NG use was 79.5 days. All studies reported a greater short-term weight gain for patients with NG than for patients fed per os, with 30% of non-adherent patients (interference with the tube or the feeding pump). NG could decrease bingeing/purging behaviours and improve cognitive functions and psychiatric comorbidities such as anxiety and depression symptoms. Results concerning the physiological tolerance of NG (digestive disorders), safety (partial symptoms of refeeding syndrome) and the psychiatric outcomes are confusing and should be taken with caution because of the many methodological limitations.

One systematic review [52], investigating the efficacy of enteral nutrition (EN) in the treatment of eating disorders included 22 studies and 1397 AN patients, 97.4 % of whom were females. One study concerned only hospitalized adolescent boys [110]. The nineteen studies evaluating the use of enteral nutrition in the treatment of anorexia nervosa, reported a significant short-term weight and/or BMI gain, but results were more uncertain in the long term. Five studies evaluated the characteristics and outcomes of the use of enteral nutrition in the treatment of binge-eating/purging behaviours, among which four studies were conducted in home settings [108,109,111,112]. The combined results of these studies confirmed that transient exclusive EN use decreased the frequency and severity of bingeing/purging behaviours. Three studies [113,114,115] on severe AN patients with BMI ≤ 11.5 reported that EN was initially better accepted than oral intake, and that EN is a safe and well-tolerated therapeutic strategy for high-risk patients. No major side effects in comparative studies were reported concerning transient hypophosphatemia, well controlled by biological monitoring, and transient and moderate digestive disturbances were resolved with treatment. Most studies reported various, transient EN interference strategies, without massive refusal for the reinstatement of tube feeding. Hale et al, discussed the limitations of the study, including various selection biases and ethical limitations for the conduct of blind randomized trials in the EN clinical context.

##### Oral Nutritional Supplementation

High-calorie liquid supplements can be prescribed to supplement oral food intake or to substitute for calories refused in meals, to increase energy intake and to promote weight gain [106]. Different types of oral supplements varying in flavour, volume, and nutritional composition exist, and need to be adapted to individual therapeutic purposes. Evidence is really scarce in the field of AN.

We found no systematic review on this topic, but benefits and adverse effects for high-energy liquid supplements among feeding methods are reported by Hart et al. [116] with the combined findings and conclusions of five descriptive studies [117,118,119,120]. Oral nutritional supplements can meet the high calorie requirements for weight gain in a smaller volume (125–300 mL) than food, and can thus be helpful for patients with digestive discomfort and/or for vulnerable patients in avoiding early satiety. This can lead to a better and faster nutritional recovery, and a reduction of hospital stays by shortening the duration of treatment. The main adverse effect is the risk of addiction to oral supplements creating an obstacle to food reintroduction, by reinforcing avoidance behaviours or by encouraging dependence on artificial food sources. These findings suggest that oral nutritional supplements can be considered as a part of dietary and medical care, and should be administered with precise and specific objectives explained to patients and integrated into a multidisciplinary approach.

##### Parenteral Feeding 

Parenteral feeding is contraindicated in anorexia nervosa because of the major risk of metabolic and infectious complications [45].

##### Micronutrient Supplementation

No systematic review was found on this question. Several micronutrient deficiencies (including vitamins, minerals and trace elements) are described among patients with eating disorders [121]. These deficiencies are the consequence of restrictive food and low micronutrient intakes widely described among eating disorder patients [122]. Among malnourished patients, initial asymptomatic electrolyte, vitamin and trace element deficiencies can often worsen with re-feeding because of the increased needs, and lead to the occurrence of refeeding syndrome (RS) [45]. Prophylactic electrolyte and micronutrient supplementation is recommended for eating disorder patients with high risk of RS by the French and American guidelines on eating disorders [2,3], especially in long-lasting renutrition among adult AN patients with severe under-nutrition and a very low weight. This supplementation, in addition to unspecific vitamin and trace element supplementation, includes phosphorous (0.5–0.8 mmol/kg/d), and thiamin (200–300 mg/d) [105,123]. 

##### Zinc 

Zinc deficiency is frequent in AN patients. Zinc is reported to be an appetite stimulator and to improve depression and anxiety. Zinc increases the expression of NPY and orexin m-RNA in experimental animals and plays a role in limiting the progression of cachexia and sarcopenia [124]. 

One RCT study [125] evidenced a BMI increase that was twice as rapid and an enhancement of brain neurotransmitters, including gamma-aminobutyric acid (GABA), in the group receiving zinc supplementation. No side effects are reported. Birmingham et al [125], suggest that daily oral supplementation should be considered for malnourished AN patients.

##### Vitamin B12 and Selenium

Other rare micronutrient deficiencies are reported, such as cases of sensory neuropathy resulting from vitamin B12 deficiency [126] and cases of cardiac involvement resulting from selenium deficiency [127]. There are no recommendations on specific supplementation with vitamin B12 or selenium. However, a plasma concentration assay should be performed, and supplementation should be administered if any specific clinical or biological symptoms are observed in severely malnourished AN patients.

##### Polyunsaturated Fatty Acids (PUFAs)

Polyunsaturated fatty acids (PUFAs) including essential fatty acids, linoleic (n-6) and alpha-linolenic n-3 (n-3) acids, and long-chain fatty acids (LC n-PUFAs), seem to provide different benefits for various neurological and psychic disorders by acting on the brain and the inflammatory system [128].

A recent comprehensive overview of the literature [129] reported that AN patients have modified PUFAs levels. Shih et al [129], reported on one case [130] and two cases series [131,132] concerning the effectiveness of polyunsaturated fatty acid supplementation and concluded that polyunsaturated fatty acids and particularly n-3 and n-6 PUFAs could be a novel adjunct medication for AN patients to treat food aversion, comorbid anxiety and depression and promote weight restoration.

#### 3.3.2. Functional Digestive Disorders 

Functional Digestive Disorders according to the Rome III criteria are common in anorexia nervosa [133,134,135]. Reported lesions are dysphagia and gastric burns, described respectively in 6% and 22% of patients, with no clear link to structural involvement of the oesophagus [136]. In a prospective cohort study including inpatients with eating disorders, 96% reported postprandial fullness, 90% reported abdominal distension and more than half complained of abdominal pain, gastric distension, early satiety and nausea [137]. Classic therapies are not very effective and there are few studies on the subject. Refeeding inducing a return to normal weight, remains the most effective therapeutic option; however no systematic review exists on this topic specifically in AN. We report here, empirical data concerning therapies provided in digestive disturbances associated with AN. 

##### Drugs Acting on the Gastro-Oesophageal Cardia and Gastric Motility

According to the current guidelines of the American College of Gastroenterology [138], the prokinetic agents of choice are metoclopramide, erythromycin, azithromycin, and domperidone [139]. 

Metoclopramide is the first-line drug (moderate recommendation, moderate level of evidence [138]). It is a dopamine D-2 receptor antagonist that acts by stimulating the parasympathetic innervation of the stomach to increase the motility and contraction of the smooth muscles of the stomach. It should be started at a low dose, 2.5 mg 30 min before meals. This prescription should be monitored clinically because of the risk of acute dystonia and the cardiac risk with the prolongation of the QT interval. 

Erythromycin is an antibiotic that also works at low doses, as a motilin agonist, which is a stimulant of gastric peristalsis. It has a prokinetic effect that improves the symptoms of gastroparesis. However, erythromycin has non-negligible adverse effects, and its effectiveness is limited to a few weeks because there is a saturation effect of the receptors. Long-term use can induce a decrease in the response to the drug (strong recommendation and a moderate level of evidence). Moshiree et al, have also shown that azithromycin has a similar motilin agonist effect to erythromycin and can be prescribed at a 250 mg daily dose [140].

Domperidone is a D-2 dopaminergic receptor agonist similar to metoclopramide with fewer central nervous system side effects (moderate recommendation and a moderate level of evidence) [138]. Due to the serious cardiac side effects, domperidone is subject to recent restrictions on use, particularly among underweight AN patients.

##### Other Drugs for the Gastro-Intestinal Tract

Trimebutine, a smooth muscle relaxant, can be useful in treating irritable bowel syndrome, particularly during the initial re-feeding period [141].

Proton pump inhibitors are often administered to AN patients in a context of gastroesophageal reflux or during tube re-feeding, and they are the first-line treatment because of their efficacy and supposed safety [142]. In fact, proton pump inhibitors have various potential side effects involving bone and renal and digestive functions, and they can interact with psychotropic medications in AN patients [143].

##### Laxatives 

In the context of constipation in AN, the use of laxatives should be evaluated for the risk-benefit balance, and non-irritant osmotic laxatives should be proposed in priority in association with a gut muscle relaxant such as trimebutine. Indeed, they are not very effective, and there is a significant risk of abuse as a strategy for weight control. Their use should be cautious because they can lead to dehydration or hypokalaemia [144], and a progressive withdrawal is necessary to limit sub-occlusive risk.

##### Probiotics

The gut microflora contributes to the regulation of feeding behaviours and probably has a significant impact on the regulation of responses to stress [145]. Recent findings support the concept of altered host-microbe symbiosis in patients with AN, which could be one of the key factors in the pathophysiology of AN. Probiotic gut microflora modulation could be an interesting biotherapeutic strategy [146,147], but currently no data exists.

To sum up, digestive symptoms are common in patients with AN, they are a source of physical and psychic complaints, and can be a barrier to re-feeding. Functional digestive disorders should be appropriately managed using specific medications restricted in time to relieve patients and facilitate their adherence to the oral or enteral feeding program. Despite the lack of data on their efficacy in AN, these drugs should be considered as an adjunctive therapy on a case-by-case basis among patients with severe functional digestive symptoms, and their relevance should be regularly reassessed. 

#### 3.3.3. Endocrine Medications 

Anorexia nervosa is associated with numerous neuroendocrine dysfunctions associated with modified plasma hormone levels and blunted, suppressed or paradoxical responses to dynamic tests, involving the hypothalamic-pituitary-gonadal growth hormone (GH)-insulin-like growth factor-I (IGF-I) and the hypothalamic-pituitary-adrenal axis, thyroid function, several adipokines, such as leptin, gut peptides such as ghrelin and YY peptide, and the posterior pituitary (oxytocin and anti-diuretic hormone). Endocrine disturbances can generate severe and irreversible complications involving osteoporosis, puberty, fertility or growth and can in addition perpetuate AN symptoms and psychiatric comorbidities [148,149]. 

Among AN patients, the majority of endocrine disturbances are attributable to weight reduction and to the low energy availability as a result of chronic starvation, but also due to neuro-psychic alterations, and consequently, the key treatment consists in weight restoration and in treating psychic disorders. Since hormone changes can also act as maintenance or aggravating factors on AN cognitive and behavioural symptoms, on psychiatric comorbidity (anxiety, depression) and on neuro-psychic function, it seems essential not to neglect endocrine comorbidities and to possess an adequate range of specific medications.

To date, no systematic review concerning endocrine medications exists, but we found a few recent, innovative studies on promising hormone treatments conducted among female AN patients: one study concerned recombinant human growth hormone (rhGH) replacement among adult AN women [150], one study concerned rhGH treatment among AN children [151], one study concerned oestrogen replacement among adolescent AN girls [152], one study concerned GnRH among weight-recovered AN [153] and one study concerned recombinant human leptin among underweight women [154]. We report these five studies on these innovative hormone medications in the following sections, and they are summarized in Table S1. All other studies about endocrine medications and bone health and osteoporosis were reported in previous systematic reviews and are summarized in Table 3. 

##### Growth Hormone (GH)-Insulin-Like Growth Factor-I (IGF-I) Axis Medication

Nutritionally acquired resistance to GH, with high levels of this hormone and a disruption of the circadian dynamics of GH secretion, high levels of the GH secretagogue ghrelin, and low serum IGF-I levels have been reported among young AN patients [20]. The pathophysiological mechanisms underlying pubertal delay or arrest and low height velocity (HV) are complex during the critical window for the pubertal growth spurt. These mechanisms can affect adult height, but they are still incompletely understood. After the patients’ nutritional and mental state has improved, catch-up growth is highly variable, from complete catch-up to a complete failure to gain height [155,156]. About one third of girls with severe early-onset AN are at risk for adult height deficit [157]. It remains unclear whether the high rates of associated psychiatric comorbidities, such as depression and anxiety, contribute to hypercortisolemia and persistent severe growth deficiency. It can also be noted that among children and adults, GH and IgF1 have various metabolic effects on body composition and trophic effects on bone formation and osteoblastic activity [150]. 

In a randomized placebo-controlled study, Fazeli et al [150], showed that supraphysiological rhGH administration for AN adult women for 12 weeks failed to increase IgF1 levels, but significantly decreased the total fat mass and fat mass percentage (rhGH, −2.5 ± 0.6%, vs. placebo, 2.2 ± 1.1%; *p* = 0.004) and leptin levels in the rhGH group. Glucose, insulin, free fatty acid levels, bone markers (N-terminal propeptide of type 1 procollagen, type I collagen C-telopeptide), and weight did not differ between the two groups. These results support the independent metabolic roles of GH and IgF1 and the fact that supraphysiological rhGH is not a useful medication for adult AN women because of the negative effects on nutritional status via increased lipolysis, and on gonadal function via the effects of leptin. 

In a proof-of-concept study reported by Léger et al [151], recombinant human growth hormone (rhGH) treatment has recently been shown to greatly increase HV among AN adolescents with delayed puberty and prolonged severe growth failure (HV < 2.5 cm/year for at least 18 months at the age of 13.3 ± 1.1 years) within one year of treatment instatement. Serum IGF-I levels increased to the mid-normal range for all patients; HV increased significantly, from a median of 1.0 (0.7–2.1) to 7.1 (6.0–9.5) cm/year after one year (*p* < 0.002). This increase in HV was also maintained in subsequent years and adult height (−0.1 ± 1.0 SD) was close to target height after 3.6 ± 1.4 years of rhGH. The treatment was well tolerated. Despite a substantial increase in body mass index (BMI) before the start of GH treatment, mean BMI SDS did not normalize entirely. These data indicate that the increase in HV observed in these patients was probably related to hGH therapy, with only a small potential contribution of the improvement in nutritional intake and BMI. To determine whether hGH therapy should be considered an appropriate option for AN adolescent patients, a randomized placebo-controlled study evaluating the effect of hGH treatment on growth, metabolic parameters, bone mineral density and overall course of the illness in this rare and severe condition in children is currently being conducted. 

##### Hypothalamic-Pituitary-Gonadal Axis Medication

AN patients present functional hypogonadotropic hypogonadism including low levels of gonadal hormones (estradiol/testosterone), prepubertal patterns of gonadotropin hormones (Follicle Stimulating Hormone (FSH), Luteinizing Hormone (LH), reduced GnRH pulsatility with menstrual disorders in women, and fertility and sexuality disorders in both sexes [149]), although the literature on endocrinopathies among AN males is sparse [158]. Weight restoration is a crucial issue for gonadal function recovery, but individual BMI targets and time lapses to menstrual resumption are highly variable [159], and the indication for hormone replacement to restore menstrual function, and the efficacy of fertility-stimulating treatment among weight-recovered anorexic female patients, are frequently questioned. The potential impact of oestrogen on cognitive function among AN women following adolescent onset has recently been suggested [160]. 

One double-blind RCT reported by Misra et al [152] on 72 AN adolescent girls with an 18-month follow-up evaluated the impact of transdermal 17 ßestradiol (100 µg twice/week)/ 2.5 mg medroxyprogesterone acetate J1-J10/month) on anxiety, eating attitudes, and body image. Oestrogen replacement was linked to a decrease in anxiety trait scores evaluated on the Spielberger State-Trait Anxiety Inventory for Children (STAIC-trait scores) without impacting anxiety state scores (STAI-state). There was no effect of oestrogen replacement on eating disorder symptoms evaluated on the Eating Disorder Inventory (EDI II) or the Body Shape Questionnaire (BSQ-34 scores). BMI changes did not differ between groups. Oestrogen replacement leads to a reduction in trait anxiety among adolescent girls with AN that is independent from weight changes. However, oestrogen replacement did not directly impact eating attitudes and behaviours, body shape perception, or state anxiety. These results, to be confirmed, raise interesting questions and call for future research to confirm the impact of various oestrogen replacement therapies on cognitive functions, anxiety and depressive symptoms in AN.

One retrospective observational monocentric study reported by Germain et al [153], compared response to gonadotropin-releasing hormone therapy (GnRH) with 20 µg/90 min/four weeks induction cycles (repeated if there was no pregnancy) administered by a sub-cutaneous infusion pump to 19 weight-recovered AN patients (Rec-AN) (BMI > 18.5) and to patients with other causes of hypothalamic amenorrhea, including primary hypothalamic amenorrhea patients (PHA) and secondary hypothalamic amenorrhea patients (SHA). The study results reported higher estradiol and LH levels during induction cycles among Rec-AN patients than in the PHA and SHA groups; follicular recruitment and the ovulation rate were higher among Rec AN patients than among PHA patients, but similar to SHA patients; the cumulate pregnancy rate was 74 % for Rec-An (vs 73% for SHA et 14% for PHA). No adverse side effect and no excessive response to stimulation were reported. This study showed that pulsatile GnRH therapy could be a safe and efficient treatment in hypothalamic amenorrhea among weight-recovered AN patients. 

##### Leptin

Previous animal model studies reported that leptin, an anorexigen adipokine regulating LH pulsatility, gonadal function, puberty development and fertility, could participate in starvation-induced amenorrhea among AN patients [161]. Leptin levels are decreased in malnourished AN patients, and recent research discussed the potential interest of recombinant human leptin treatment, which could normalize reproductive hormones and restore gonadal function among female AN patients, and the interest of combining recombinant human leptin with oestrogen therapy [162]. 

One prospective study, reported by Welt et al [154], concerned the use of recombinant human leptin (r-metHULeptin) for up to three months among eight secondary hypothalamic amenorrheic adult women (18–33 years) versus six amenorrheic control subjects. Amenorrhea among the treated patients was secondary to recently increased physical exercise or weight loss but without AN diagnosis. The study reported increased LH pulsatility after two weeks of treatment, and increased estradiol levels and ovarian activity, over a period of three months among treated subjects. Levels of thyroid hormones (free T3), IgF1 and IGF1-BP3 and osteoformation biomarkers also increased with leptin treatment. The safety and tolerance of leptin administration and the impact on eating behaviours among AN patients are in debate and require further research. 

There are serious gaps in knowledge and no approved treatment for gonadal deficits or other endocrine dysfunctions in AN despite the various severe consequences on somatic, nutritional and psychiatric aspects of the low levels of reproductive hormones and other hormonal disturbances such as prolonged hypercortisolism. Recent studies suggest interesting new approach strategies, such as sexual hormone therapy to normalize oestrogen deficit, and restore puberty and fertility processes, with a potentially positive impact on cognitive functions, mood, anxiety, and bone health, and reduced spontaneous fracture risk. To date, there is no data concerning gonadal function treatments for male AN patients.

#### 3.3.4. Bone and Osteoporosis Medication

Anorexia nervosa leads to a loss of bone mass, accompanied by low bone mineral density (BMD), secondary osteoporosis and increased fracture risk, as a result of malnutrition and hormonal imbalance [163]. Almost all adult AN patients (92%) have a BMD 1 SD below controls and 38% of patients have a BMD 2.5 SD below controls. For the same duration of amenorrhea, AN patients who develop AN during adolescence have lower BMD than those who present AN later in adult life. As suggested by Misra et al, long-term use of SSRIs could contribute to low bone mass in AN [164]. Given the impact of AN on bone metabolism, fracture risk should be assessed in AN patients using dual energy X-ray absorptiometry (DXA). The U.K. National Osteoporosis Society (NOS) recommends monitoring BMD by DXA every two years.

We found four systematic reviews concerning the treatment provided for bone health and osteoporosis [39,44,49,50]. One of them concerned the impact of weight gain/restoration on bone [44], one was about impact of oestrogen replacement on bone [39], and the two other studies concerned the impact of various somatic medications on bone health [49,50] We report these four systematic reviews in the following sections on the basis of the therapeutic strategies provided.

##### Weight Gain/Restoration

Restoring weight and normal function of the gonadal axis (with restoration of menses for women) is one of the goals of the treatment of AN and is essential for bone health. 

In a systematic review [44] which included 18 studies with follow-up periods from 12 to 90 months of female adolescent AN patients, eight studies showed no significant change in BMD after weight gain/restoration (follow-up 12 months), one study showed decreased BMD after weight restoration (follow-up 12 months), and nine studies showed BMD improvements with weight gain (mean follow-up 30 months) without total catch-up with controls. Therefore, there is strong evidence for at least a stabilization of BMD with weight gain and/or weight restoration. Longer follow-up (more than 12 months) provides evidence of an increase in BMD with weight gain. Nonetheless low BMD and fracture risk persist after weight recovery. In an adult study [165] the patients whose menses were restored had an increase in spine BMD independently from weight restoration, and weight gain improved hip BMD, whereas the patients with no weight gain nor restoration of menses had an annual rate of BMD decrease of 2.5%. Weight gain and restoration of the gonadal axis can be difficult to obtain in clinical practice. In addition, BMD is persistently low a long time after recovery for AN patients without complete catch-up with weight and menses restoration [166]. The adjunction of complementary medications to treat low BMD and limit fracture risk among pre-menopausal AN women needs to be considered. Only one study [167] on male adolescents with AN was reported, evidencing a rapid and positive bone density evolution in case of weight restoration.

##### Oestrogen Replacement Therapy

Oestrogen inhibits bone resorption and hypogonadism resulting from food restriction in AN and contributes to increased bone resorption as a result of hypoestrogenia in women. 

One cross-sectional study [168] has shown a higher spine BMD for AN subjects exposed to oral contraceptives (OC) combining oestrogen and progesterone compared to AN patients without OC, but Elkazaz et al. [169] reported that healthy premenopausal women with OC current use have a lower BMD compared to women with past OC use and/or non-use, and that this relationship seems in part mediated by IGF1 suppression by oestrogen and thus should not be recommended as a bone accrual medication for adult AN patients.

One systematic review [39] including 10 studies with eight placebo-controlled RCTs evaluated the influence of oestrogen therapies on bone mineral density (lumbar spine and femoral neck or hip BMD by DXA) among adolescent and premenopausal adult women with amenorrhea [39]. Five RCTs used oral contraceptives and three RCTs used hormone replacement therapy. The results were poor quality and generally disappointing regarding oral contraceptives with various ethinyl estradiol doses administered (20–35 µg/d) and various combined progestins, small samples and short follow-up. Of the five studies using oral contraceptives, only one reported increased BMD in the lumbar spine [170], while in the other studies bone loss was not modified or continued to progress. Lebow et al concluded that oral contraceptives were poorly effective in treating bone loss among adolescent or young adult amenorrheic AN patients [39]. Physiological hormone replacement therapy yielded more interesting results, particularly with one study reported by Misra et al, [171] on physiological transdermal oestrogen (17ß estradiol) replacement therapy among AN adolescent girls (with incremental doses of oestrogen mimicking oestrogen pubertal secretion for the youngest patients and 100 µg patches for bone-mature patients) combined with cyclic progesterone. This increased hip and spinal BMD, with an increment comparable to the healthy control group, in a well-designed randomized controlled 18-month trial, but it did not provide complete recovery at the end of the trial, as BMD was still lower in the AN group than in the control group [171]. Therefore, transdermal oestrogen replacement therapy, by bypassing the IgF1 suppressive effect of oral oestrogen, could be recommended for adolescent females suffering from AN.

##### Hormonal and Other Somatic Medications

One systematic review [49] concerning 20 studies (10/20 were double-blind RCTs) reported a synthetic assessment of the effectiveness of weight restoration and interventional studies exploring various somatic drugs (oestrogen replacement therapy, recombinant h-GH, recombinant h-IgF1, DHEA, biphosphonates, teriparatide) on bone health among adolescent and adult AN women.

The most recent systematic review [50] included 19 studies (10/19 were double-blind RCTs) and concerned adjuvant medications potentially active on bone, such as various oral contraceptives (OC) containing Ethinyl Estradiol (EE) (EE or EE/levonorgestrel or EE/progestin or EE/norgestimate), various oestrogen replacements (transdermal 17ßE/progesterone or oral EE/progesterone), teriparatide (TPt), alendronate, rhIgF1, menatetrenone (MED) (vitamin K2), risedronate and transdermal testosterone.

The results from these two systematic reviews concerning various medications and pharmacological interventions are summarized in the sections below.

##### Bisphosphonates

Biphosphonates inhibit osteoclast activity and bone resorption, they increase BMD and reduce fracture risk in post-menopausal women. Three trials have explored the impact of bisphosphonate treatment on BMD in AN (one RCT with alendronate [172], two with risedronate: one controlled trial [173] and one RCT [174]). After 1 year of alendronate in an adolescent AN group (baseline Zscore ≤ −1), weight was the main determinant of BMD. After controlling for body weight, alendronate increased femoral neck BMD. This does not provide sufficient proof to support the use of alendronate in adolescent osteoporosis in AN.

Among adult premenopausal AN patients with osteopenia (Tscore of −2.7 ± 2) [173] spine BMD had increased by 4.9±1% after nine months of treatment with risedronate compared to a decrease of 1 ± 1.3% in the control group. After one year of risedronate [174] (baseline Zscore −1.5 ± 0.7) spine BMD increased by 3.2%, whereas it was unchanged in the placebo group. 

Oral bisphosphonates have been associated with upper gastrointestinal tract ulcerations. Nonetheless, in the three trials in AN no adverse effects on the gastrointestinal tract were reported. Intravenous bisphosphonates could be an option in AN, but they have yet to be tested in this population. The main question concerning the use of bisphosphonates in AN resides in its long half-life and its potential harm to the foetus in case of pregnancy. For the time being, for premenopausal adult AN women, the prescription of bisphosphonates cannot be widely recommended, and individual prescription should be discussed on a case-by-case basis.

##### Testosterone

In the studies using transdermal testosterone administration in women with AN there was no significant changes in markers of bone formation, and no increase in spinal BMD when transdermal testosterone was administered without risedronate [174,175].

##### DHEA

The sole significant effect of DHEA was observed when combined with oral contraceptive (20 µgEE/ 0.1 mg levonorgestrel) with a stabilisation of femoral neck BMD [176].

##### IgF1

RhIGF-1 replacement increased bone formation markers in both adolescents and adults AN patients [177,178,179]. Effectiveness of combined oral contraceptives or 17ß estradiol replacement and rhIgF1 are discussed [50].

##### Teriparatide

Teriparatide is a human recombinant parathyroid hormone, anabolic on bone and it is recommended for the treatment of post-menopausal osteoporosis. A six-month RCT [180] studied the impact of teriparatide among older AN patients (mean age 47, Tscore ≤ 2.5), and evidenced that teriparatide increased spine BMD by 6% (+ 0.2% in the control group). This result supports the use of teriparatide as anabolic agent for older AN patients.

##### Menatetrenone (MED) (Vitamin K2)

Administration of MED (vitamin K2) in AN among Japanese women over a nine-month period reduced bone loss, but there was no increase in BMD [181]. 

Evidence-based data from these recent comprehensive studies suggests that the safest and most effective strategy to protect and improve bone density in AN and prevent fractures among adolescent and premenopausal women is restoration of weight with the resumption of menses. The most promising available medications include 17 ß estradiol replacement (such as the transdermal estradiol patch) for adolescents and bisphosphonates for adults. 

##### Vitamin D and Calcium Supplementation

Low levels of vitamin D and inadequate calcium intake are associated with increased fracture risk and low BMD. In addition, D3 hypovitaminosis could be responsible for the lack of inflammatory response and depressive symptoms among patients with long-term eating disorders. Calcium intake among adolescent AN patients has been described as comparable to that among controls, partly as a result of supplements [182]. While their intake and the bioavailability of oral ergocalciferol among young AN patients was similar to that of healthy controls, AN patients have lower serum levels of 25 and 1,25OH-Vitamin D [183]. In addition, patients who have lower serum levels of vitamin D (<20 ng/mL) have lower hip BMD [184]. After weight gain, the spine BMD increase was greater in the group of patients with higher serum vitamin D levels (≥30 ng/mL) [185], supporting the use of oral vitamin D supplements to obtain sufficient serum levels during weight gain. No RCT prospective trial has been performed to evaluate the efficacy of calcium and vitamin D supplements alone on BMD among AN patients. Nevertheless, given the impact of calcium and vitamin D on bones, although the efficacy of calcium and vitamin D supplementation has been poorly evaluated in AN, we recommend a total intake of calcium of approximately 1000–1200 mg and vitamin D supplements if serum levels of vitamin D are insufficient or if there is a secondary hyperparathyroidism [186]. Since oral calcium supplements can be associated with an increased risk of incident coronary atherosclerosis, a calcium-rich diet should be privileged if possible among AN patients [187]. 

## 4. Discussion

The original aim of this multidisciplinary overview was to summarize all the literature published about the use of medication in the psychiatric, somatic and nutritional aspects in AN. 

Evidence based on the efficacy of medication in anorexia nervosa is scarce whether for the psychological, somatic or nutritional sphere. The evidence base is sparse, as the literature reports mainly case reports, cases series, open studies and some RTCs. In addition, for the RCTs, methodologies have numerous failings such as the heterogeneity of study designs, research methods, population samples, and intervention modalities [6,37,44,45,50]. The aims of the published research were initially to find medication that would cure AN by overcoming the key resistant symptom, the need for weight gain. Many drugs have been tested in AN on the rationale of their side effect in terms of weight gain in other disorders (for example lithium, antihistamines, cannabinoids, etc.) or their action on clinical manifestations observed in AN (depression, anxiety, obsessions, hyperactivity). In parallel, some psychotropic drugs have been tested in connection with hypotheses of neurobiological etiopathogenic neuro-transmitter involvement in the development or maintenance of AN (for example noradrenalin and serotonin for antidepressants, dopamine for antipsychotics). Recently, new neuro-hormones involved in the regulation of dimensions altered in AN, such as appetite regulation (ghrelin) or social interactions (oxytocin) have been evaluated. However, oxytocin and ghrelin have not proved their efficacy nor their safety in AN. In relation to its anorexigen effect, oxytocin needs more evaluation in AN setting [188]. These neuro-hormones should not be used in routine care practice. 

### 4.1. Somatic and Nutritional Aspects

Concerning somatic aspects, if weight gain is a crucial step to correct the majority of adaptive and functional changes resulting from undernutrition, somatic drugs have been explored from the late 1990s. The evidence of a higher risk of osteoporosis and spontaneous fracture among AN subjects with amenorrhea [17,19] raised the question of the use of bisphosphonate or oestrogen replacement to treat low bone density [39,49,50]. Other hormone replacement therapies for the endocrine consequences of AN, such as growth or pubertal delay, appeared in the 2000s in association with endocrine-paediatric management in the context of a multidisciplinary approach [151,157]. Two recent narrative update reviews on endocrine mechanisms and repercussions in AN report on available treatments and innovative therapeutic strategies in endocrinology [189,190]. Physicians’ interest in refeeding modalities [191], relating to optimal daily calorie requirements [42], the risk of over-feeding and that of under-feeding, high calorie oral supplementation [116], vitamin supplementation and functional digestive disorder medications [139], is fairly recent and corresponds to the development of specific nutritional and dietary expertise, alongside the publication of international guidelines [2,3]. Finally, in the last 20 years, we have observed a paradigm shift concerning enteral tube feeding, considered initially as unethical and coercive, and now viewed as efficient, safe and well tolerated [53]. Promising somatic medications for new targets have developed recently, with micronutrients such as zinc, polyunsaturated fatty acids (PUFAs) used in refeeding, physiological oestrogen replacement or teriparatide to treat bone health, leptin to restore gonadic function, reproductive hormone for hormonal deficits and neuropsychic functions, and GH for severe growth retardation among prepubertal adolescents.

### 4.2. Psychotropic Drugs

Since the 1960s nearly all classes of psychotropic drugs have been tried in AN [45]. Psychotropic drugs were initially compared to placebo without psychotherapy, with very poor results, but the most recent studies now use psychotropic drugs as additional treatment to global treatment approaches including psychotherapy (individual and/or family therapy). This type of use stems from an international consensus, as attested by the NICE guidelines (the most recent international guidelines published) stipulating that medication cannot be considered as the sole treatment for Anorexia Nervosa (see Table 1). 

Despite the evolution and improvement in study design, evidence-based data is scarce and possibly even less robust than it was in the WFSBP in 2011 [6] since atypical antipsychotics seem not to exhibit the efficacy they initially appeared to have.

The RCTs generally evidence negative results concerning weight, and contradictory results concerning eating disorder symptoms and psychiatric dimensions. This situation can be explained by discrepancies in study methodologies [45,48], including many limitations such as relatively short durations of treatment (weeks, or two to three months mainly) and the small sample sizes (fewer than 100 in RCTs, and generally fewer than 30). That said, one of the most important limitations to efficacy in AN is non-compliance with medication treatments: patients frequently do not take their medication. 

This situation of a poor evidence base in the existing literature is discouraging [48]. It has led, in the more recent reviews published, to giving prominence to emergent or promising interventions and recommendations for research [24,40,45,51].

In this situation, if we refer only to the evidence-based elements, psychotropic drugs should not be prescribed in AN. However, the reality in the field is quite the reverse. All psychotropic drugs are widely prescribed to both adults and adolescents with AN, and this seems to be increasing [7]. In addition, more than half of the patients have more than one psychotropic drug prescribed simultaneously. Also, dangerous drugs such as bupropion or tricyclics, are still prescribed despite the conclusions of the literature [7]. The same observation has been made in other studies on adults and adolescents in different countries [7,8,192]. These papers highlight the disparity between research conclusions and clinical practice in the treatment of AN. This underlines the importance of consensual guidelines incorporating evidence-based data. We also need expert consensus and training for practitioners on this question, especially for ED specialists, who mostly base their practice on their experience and who seem more resistant to using guidelines than general practitioners [7]. Our “experience” is the result of a complex combination of elements. It is based on core beliefs derived from different sources, including, of course, evidence-based data. Evidence can be biased in favour of drug efficacy by the publication of positive results only [7]. In addition, some studies (or their coordinators) are sponsored by the industry and their results can be biased by conflicts of interest. This last point leads the latest NICE guidelines to exclude studies sponsored by the industry from the studies analysed to establish the evidence base retained for the guidelines [4]. In addition, our experience is also impacted by psychiatric theoretical orientations, [7] or good or bad experiences with one or several patients.

### 4.3. Use of Psychotropic Medication in AN

Once we have discussed these elements, the central question for this review is still unresolved: how should we use medication in AN? 

As recommended by most guidelines, we need to evaluate the global situation of the patient, including psychiatric, medical, nutritional and social aspects, to propose the best-suited treatment. Medication should be prescribed on the basis of the clinical evaluation. This evaluation should include the patient’s opinion about the treatment [4,24].

Concerning psychotropic medication, comorbidities (including mood disorders, anxiety disorders, obsessive disorders) should be evaluated and treated [18,45] if they are real disorders and not “artefacts” that will improve with re-feeding [193]. These disorders are usually treated by teams on the assumption that treating anxiety and depressive disorders facilitates AN treatment, or at least improves their quality of life [45]. These disorders are usually treated according to the specific recommendations for each, and taking into account the particular risks of side effects resulting from low weight and metabolic disturbances (possible depletion in potassium, phosphorus, magnesium, and zinc), and somatic complications concerning hematologic, renal, hepatic and heart functions that can interact with medication. Antidepressants and atypical antipsychotics are the most frequently evaluated drugs and the most widely used in practice. 

Chlorpromazine is no longer used for AN because of its severe adverse effects, including seizures; low doses of haloperidol could be effective as an adjunct treatment for patients with severe, treatment-resistant conditions with marked self-image alteration [48]. Atypical antipsychotics can sometimes be useful to help patients with AN, in case of high levels of anxiety [75] or in adults with a long illness duration [76], but practitioners should be aware that they prolong the QT segment and can be dangerous in case of malnutrition by leading to cardiac death associated with ventricular arrhythmia. Their prescription should be monitored for safety, including regular electrocardiogram and ionogram assessments. In addition, because of the risk of metabolic syndrome, such as hyperglycaemia, dyslipidaemia or HTA, the usual guidelines for the monitoring of antipsychotics should be applied. Benzodiazepines are also widely used and their use is questionable, especially among young people, as they induce dependency and can lead to abuse or misuse [7].

A psychotropic drug can be useful to manage eating disorders or psychiatric symptoms. Himmerich et al. [194] in a recent survey among AN patients and carers and reported that people with anorexia nervosa want medication to help with anxiety and sleep problems [24]. However, the specific treatment of AN also requires re-feeding, and/or cessation of binging and purging, specific psychotherapy (individual or family therapy) and work on the social impact of the illness. Medications, and especially antidepressants, are less effective among those that are acutely ill and underweight [18]. 

Outside situations of emergency (suicidal ideas or risk, very severe anxiety or OCD), following the discussion with the patient, it is possible to wait a few weeks in order to later reassess the need for specific treatments for possible comorbidities [195]. In order to diagnose whether psychiatric symptoms are linked to a comorbid diagnosis or to malnutrition, it is useful to investigate the personal and family history of comorbid disorders and the chronology of appearance of AN and psychiatric symptoms. Diagnosis should be based on symptoms, their evolution, the chronology of onset and individual and familial history of comorbidities [18].

A general discussion with the patient (and the parents for a minor) is necessary to review side effects, risks, benefits, and alternatives. One important point to make is that psychotropic drugs do not induce weight gain in AN but can alleviate negative psychological symptoms. This will be important for the therapeutic alliance. Practitioners should always have in mind that to be efficient, a medication needs be taken, and AN patients are often not observant, especially for antipsychotics [37]. In addition, to be efficient, a medication needs be absorbed. When patients purge after they have taken their pills or have swallowing phobia or food orality disturbances, they cannot be efficient. Both points should be discussed with the patients.

As in other reviews, we will conclude on perspectives for future research on the topic of medication in AN. 

### 4.4. Perspectives 

Several perspectives suggest the need for the development of innovative treatments. 

Part of the lack of evidence could be attributable to the heterogeneity of significant symptoms and the treatment responses encountered in AN. Very few studies thus far have stratified the subtypes or clinical features of the disorder. Anorexia nervosa is a heterogeneous disorder and a single “optimal” drug for all individuals is highly unlikely. Targeting more homogeneous subgroups could be helpful. Thus, growing evidence derived from various approaches supports a specific staging trajectory for anorexia nervosa, and there is preliminary evidence that interventions should be matched to the stage in the illness (for review see [196]). Interventions tailored according to the stage of the illness or the developmental trajectory could be valuable options. Certain other clinical features could be considered for more homogeneous subgroups of patients, since they could be associated with a specific form of the illness, or lower response to medication, for instance the AN subtype (AN-R, AN-BP, AN/B, AN/P), age (differentiating pre and post-pubertal adolescents and adults), gender, cultural environment, current and maximum pre-treatment BMI, associated personality traits, psychiatric comorbidities [45] or a history of childhood abuse [197,198]. A number of latent class and latent profile analyses have been performed on symptom and personality factors to stratify the endo-phenotypes spanning AN [197,199,200,201]. These sub-categorizations could be a starting point to homogenize samples. 

Also, there is currently emphasis on using precision medicine to identify targets, which should lead to more effective treatments. Symptoms that maintain the disorder may differ across individuals and participate in the lack of evidence. A novel methodological perspective is thus to address the extreme heterogeneity within AN and to develop and adapt treatments to each individual, which could be of considerable interest in the near future [202]. 

Finally, beyond a categorical approach, treating one or several specific dimensions associated with the illness rather than the illness overall could be useful, since changing targets might change clinical outcomes. Some salient dimensions in AN, such as delay discounting or cognitive impulse control, could potentially be targeted by drugs or a neuromodulation approach [203]. 

Concerning non-psychotropic drugs used in AN, these are mainly used to treat medical conditions associated with AN. It is critical to underline that weight gain and restoration of normal weight is the first line of approach to the management of these conditions whenever possible. Therefore, refeeding modalities for weight gain or restoration, including the specific indications for enteral nutrition, oral nutritional supplementation, micronutrient supplementation and specific drugs for functional digestive disorders, seem absolutely essential, but to date no guidelines exist on nutritional therapeutic strategies for AN.

Among the drugs available, sex hormone treatments need to be discussed, as they are fairly widespread in use, but their effects are a source of considerable debate. Various arguments should be taken into account in the assessment of the risk-benefit balance of their prescription. As demonstrated by our results, oral contraceptives have failed to show any significant benefit in protecting bones among patients with AN. Although certain issues could explain this lack of effect, such as the heterogeneity of the patients included across studies, and variable treatment and follow-up durations, the suggestion is not to use oral contraception to protect bones in AN. On the other hand, transdermal estradiol has been found to be a more physiological form of oestrogen replacement and more promising than oral administration in managing osteopenia among adolescents with AN. This explains the position of the recent British national guidelines suggesting hormone replacement therapy with 17-β-estradiol (with cyclic progesterone) rather than oral contraception, and only for young AN women (13–17 years). Furthermore, the decision to prescribe oral contraception is not without drawbacks. For example, the return of menstrual function, which indicates adequate weight restoration, is masked by the cessation of bleeding induced by contraceptives. Also, its use can provide a sense of reassurance that patients are protected against osteopenia, which can reduce efforts for weight rehabilitation. On the other hand, it is important to keep in mind that amenorrhea, occurring in 66 to 84% of women suffering from anorexia nervosa, favours the absence of contraceptive measures, explaining a particularly high rate (up to 50%) of unwanted pregnancies in this population. Finally, somato-psychic tolerance and compliance with hormonal replacement are very limited due to fat phobia, menstruation and bleeding refusal in many adolescent AN patients. Recent studies evaluated the interest of testosterone replacement therapy in AN women based on the fact that hypothalamic amenorrhea is associated with a profound androgen deficit. Transdermal testosterone replacement increases spinal BMD when administered with risedronate and can stimulate bone formation [174]. In addition, this could be an effective medication in women with treatment-resistant depression by improving depression and cognitions [175]. Otherwise, as mentioned previously, SSRIs seems to be inefficient in conditions of undernutrition, and future studies should assess optimal renutrition and adapted hormone replacement to potentiate the effectiveness of psychotropic medications [49]. To date, testosterone replacement therapy is not recommended for female nor for male AN patients. Concerning the use of medications for bone health and BMD improvement, despite the promising results of studies on sexual hormone replacement, bisphosphonates and combined interventions among adolescent and premenopausal women, the safest and most effective strategy to protect and improve bone density and prevent spontaneous fractures risk is, for now, the restoration of weight and menstrual recovery. Further studies are needed to establish standards for the treatment of osteoporosis in AN. 

There are no specific treatments taking into account particular aspects of adolescent or adult male AN patients and current proposed treatments are similar to those for AN women [158]. Specific research on the male AN population is insufficient and needs to be developed, particularly with regard to testosterone and other therapies, and the benefits of specific drugs on somatic, cognitive and psychiatric functions that can influence evolution and prognosis in AN boys and men.

### 4.5. Strengths and Limitations of This Overview

As stated by Pollock et al. (p16) [204] “overviews are a relatively new methodological innovation, and there are currently substantial variations in the methodological approaches used within different overviews”. We defined a methodology for this overview on the basis of elements from the literature, but due to the characteristic of the literature concerning medication for AN we could not totally fulfil all the quality criteria previously defined [205].

Indeed, a good quality overview should include systematic reviews with four characteristics [205]: 1. They should not substantially overlap. 2. They should focus on the precise question asked by the overview. 3. They should be high quality. 4. They should be up-to-date. 

There is considerable overlap between reviews and meta-analyses selected in our overview. In order not to bias our conclusions we focused on the more recent reviews or meta-analyses; when meta-analyses and reviews were available on the same studies we focused on the meta-analysis conclusions, and when two reviews were available we focused on the more recent one including the more recent research. We selected only systematic reviews with a well-defined methodology but methodological quality was not homogeneous.

The conclusions we have drawn are limited by the methodology of the systematic reviews included, which use different criteria and objectives, over different periods, possibly leading to different conclusions and possibly biased by their selection. In order to alleviate this limitation we developed a systematic overview using the PRISMA guidelines, mainly based on meta-analyses and systematic reviews of RCTs and open comparative studies, but not on narratives reviews, retrospective studies or case reports.

Our findings are also limited by the quality of the published literature on the topic of medication for AN, which also impacts the conclusions, since most of the studies published on the subject are open studies, retrospective studies or case series or case reports, and RCTs are rare. The reason for this situation is linked to both AN patient refusal to participate in RCTs and to the clinical context, and also to the relatively low frequency of AN. Otherwise, it is also linked to ethical reasons, particularly on the refeeding topic [52] or growth hormone replacement [151]. In addition, the existing RCTs are poor quality, as they were conducted with various methodological procedures, on heterogeneous, small samples, and over different durations of follow-up. Finally, these studies were mostly conducted over short treatment durations whereas AN is a chronic disorder. 

## 5. Conclusions

Perspectives for future research on medication in AN should include more specific phenotyping for psychological dimensions and comorbidities, also taking into account more somatic and anthropometric data, such as premorbid weight, body composition, nutritional and hormonal markers of undernutrition, so as to optimize the assessment of the efficacy of medication.

There is a need to make progress and develop innovative therapeutic strategies, especially for severe, chronic forms of AN with resistant psychiatric comorbidities, and for pre-pubertal AN patients with severe somatic prognosis, by targeting medication more efficiently through improved understanding of their etiopathogenic mechanisms [189,190]. A future perspective is to address the extreme heterogeneity within AN and to develop psychotropic treatments for the various clinical dimensions observed. We should develop new, more sensitive and specific biomarkers, especially for bone microarchitecture, because evaluating BMD is not sufficient to predict fracture risk. In addition, we need better-adapted techniques to measure the benefits of weight gain and nutritional status, including body composition analyses [176].

There are exciting perspectives for the development of transdisciplinary studies, based on well-defined and phenotyped sub-groups, including psychotherapeutic approaches and evaluating the impact of optimized refeeding modalities, hormone replacement, other somatic medications, or psychotropic drugs. 

Studies should evaluate synergistic benefits of these combined interventions on a global perspective, including renutrition, cognitive functions, somatic, and psychic outcomes over prolonged follow-up periods.

## Figures and Tables

**Table 1:** Extract from the National Institute for Health and Care Excellence (NICE) recommendations [4] for medication in anorexia nervosa (chapter 1.3) and eating disorders in general including anorexia nervosa (chapter 1.8).

Recommendations
**Medication for anorexia nervosa**
1.3.24 Do not offer medication as the sole treatment for anorexia nervosa.
Dietary advice for people with anorexia nervosa […]
1.3.21 Encourage people with anorexia nervosa to take an age-appropriate oral multi-vitamin and multi-mineral supplement until their diet includes enough to meet their dietary reference values.
**Comorbid mental health problems**
1.8.12 When deciding in which order to treat an eating disorder and a comorbid mental health condition (in parallel, as part of the same treatment plan or one after the other), take the following into account:
- The severity and complexity of the eating disorder and comorbidity.
- The person’s level of functioning.
- The preferences of the person with the eating disorder and (if appropriate) those of their family members or carers.
1.8.13 Refer to the NICE guidelines on specific mental health problems for further guidance on treatment.
**Medication risk management**
1.8.14 When prescribing medication for people with an eating disorder, and comorbid mental or physical health conditions, take into account the impact that malnutrition and compensatory behaviours can have on medication effectiveness and the risk of side effects.
1.8.15 When prescribing for people with an eating disorder and comorbidity assess how the eating disorder will affect medication adherence (for example, for medication that can affect body weight).
1.8.16 When prescribing for people with an eating disorder, take into account the risks of medication that can compromise physical health due to pre-existing medical complications.
1.8.17 Offer electrocardiogram (ECG) monitoring for people with an eating disorder who are taking medication that could compromise cardiac functioning (including medication that could cause electrolyte imbalance, bradycardia below 40 beats per minute, hypokalaemia, or a prolonged QT interval). **Substance or medication misuse** 1.8.18 For people with an eating disorder, who are misusing substances, or over-the-counter or prescribed medication, provide treatment for the eating disorder unless the substance misuse is interfering with this treatment. 1.8.19 If substance misuse or medication is interfering with treatment, consider a multidisciplinary approach with substance misuse services. **Growth and development** 1.8.20 Seek specialist paediatric or endocrinology advice for delayed physical development or faltering growth in children and young people with an eating disorder.

**Table 2:** Inclusion and exclusion criteria for the systematic overview of systematic reviews, meta-analyses and selected trials (Population Intervention Control Outcome and Study design (PICOS) criteria and other elements).

Parameters	Inclusion Criteria	Exclusion Criteria
Patients	- AN ^1^ (with or without the mention of the restrictive or binging/purging types) - Human studies - All ages	- BN ^2^, BED ^3^, other ED ^4^ - Mixed eating disorder samples (AN and any other ED)
Interventions	- Medication for AN (psychotropic or somatic or nutritional)	- Medication for refeeding complications
Comparators	- All comparison groups (placebo or active drug or treatment as usual)	
Outcomes	- All criteria linked to ED symptoms, psychiatric -and somatic symptoms, and nutritional aspects, as appropriate	
Study design	- Meta-analyses and systematic reviews with a detailed methodology, including RCTs ^5^ and/or open trials	- Narrative or qualitative reviews - Overviews - Reviews of unpublished data
Period considered	- Papers published between September 2011 (since the publication of The World Federation of Societies of Biological Psychiatry Guidelines for the Pharmacological Treatment of Eating Disorders) [6] and 30th January 2019	
Language	- English and French	

^1^ AN: Anorexia Nervosa; ² BN: Bulimia Nervosa; ^3^ BED: Binge Eating Disorders; ^4^ ED: Eating Disorder; ^5^ RCT: Randomized Controlled Trial.

**Table 3:** Meta-analyses and systematic reviews selected.

Author	Year	Method	Database	Type of Study	Participant Age	Review Period from	Review Period to	Medication
Aigner, M. et al. [6]	2011	Systematic review	MEDLINE	45 studies = 19 open or case studies 26 RCTs	All	1977	2011	Antidepressants; Antipsychotics (typical and atypical); Prokinetic agents; Cannabinoids; Antihistaminics; Naltrexone; Clonidine; Tube feeding; Lithium; Growth Hormone; Zinc
Flament, M. et al. [37]	2012	Systematic review	MEDLINE; PsycINFO	11 RCTs; if none were available (e.g. for paediatric EDs) open trials or case reports suggesting benefits; systemic reviews; meta-analyses; and guidelines	All	1960	May 2010	Antidepressants; Antipsychotics (typical and atypical); Mood stabilizers and anticonvulsivants; Prokinetic agents; Opiate agonists; Appetite enhancers
Kishi, T. et al. [27]	2012	Meta-analysis	PubMed; PsycINFO; Cochrane	8 RCTs	All	No limitation	March 2012	Antipsychotics (typical and atypical)
Lebow et al. [38]	2013	Meta-analysis	Cochrane; MEDLINE; Embase; Scopus; Web of Science; PsychINFO	8 RCTs on atypical antipsychotics (in any form, used for at least 4 weeks) compared to any control intervention on BMI, eating disorder, and psychiatric symptoms in adolescents and adults with AN Eligible studies assessed BMI before, during, and/or after treatment. We excluded studies that enrolled patients who had a primary psychotic disorder.	All	1998	November 2011	Atypical antipsychotics
Lebow, J. et al. [39]	2013	Systematic review	Not reported	10 studies = 8 RCTs, 2 prospective cohort studies	11–42.5 years	Not reported	Not reported	Estrogen therapies
Watson, T. et al. [40]	2013	Systematic review	MEDLINE; PsycINFO; The Cumulative Index to Nursing and Applied Health; Educational Resources Information Center; National Agricultural OnLine; Embase Scopus Access; Cochrane Collaboration libraries	32 RCTs	All	1960	October 2011	Antidepressants; Antipsychotics; Cyproheptadine; Recombinant human growth hormone (rhGH); Risedronate; Testosterone; Nasogastric tube
De Vos et al. [41]	2014	Meta-analysis	PubMed; PsycINFO; Embase; Cochrane Library	18 studies = (a) RCTs and (b) comparing pharmacotherapy with a placebo controlled condition and reported on (c) patients with Anorexia Nervosa and an age minimum of 12 years. Outcome was measured in (d) terms of weight gain	All	No limitation	October 2012	Antidepressants; Antipsychotics; Hormonal therapy
Rocks, T. et al. [42]	2014	Systematic review	PubMed; Scopus; Web of Science	7 observational studies	≤19 years	No limitation	May 2012	Nutrition therapy
Dold et al. [43]	2015	Meta-analysis	ClinicalTrials.gov; Clinicaltrialsregister.eu; Cochrane Central Register of Controlled Trials (CENTRAL); Embase; PubMed/MEDLINE; PsycINFO	7 RCTs second generation antipsychotics efficacy, acceptability, and tolerability in comparison to placebo/no treatment, even unpublished studies	All	No limitation	August 2014	Atypical antipsychotics
El Ghoch, M. et al. [44]	2016	Systematic review	PubMed	19 studies = 11 prospective non-controlled, 4 prospective controlled, 4 retrospective non-controlled	11–19 years	No limitation	No limitation	Weight gain and restoration
Frank, G.K. et al. [45]	2016	Systematic review	National Center for Biotechnology Information database	66 studies = 25 double-blind, placebo-controlled studies; 7 double-blind, placebo-controlled crossover studies; 5 single-blind, placebo-controlled studies; 23 open-label studies; and 6 retrospective systematic chart reviews	All	No limitation	2014	Antidepressants; Antipsychotics (typical and atypical); Mood Stabilizers: Zinc; Opiates and Cannabinoids; Benzodiazepines and Alpha 2 Adrenergics; D-Cyclocerine; Amantadine; DHEA; Ghrelin; Growth Hormone; Testosterone; Estrogen
Garber, A.K. et al. [46]	2016	Systematic review	PubMed; Scopus; PsycINFO; Clinical trials database	27 studies = 1 RCT, 6 prospective, 14 retrospective, 6 observational	13–38 years	1960	15 March 2015	Refeeding approaches
Kells, M. et al. [47]	2016	Systematic review (integrative)	PubMed; Embase; Cochrane CINAHL	18 studies = 2 RCTs, 6 retrospective, 5 cohort, 1 observational, 4 case reports	11–57 years	No limitation	May 2016	Tube feeding
Miniati, M. et al. [48]	2016	Systematic review	MEDLINE; PsycINFO	41 studies = 17 RCTs, 9 open trials, 12 case series and case reports, 2 retrospective observations, 1 single-blind RCT	Adults	January 1966	January 2014	Antidepressants; Antipsychotics (typical and atypical); Lithium; Clonidine; Cyproheptadine
Misra, M. et al. [49]	2016	Systematic review	PubMed	20 studies = 10 RCTs, 8 prospective observational studies, 1 retrospective cohort study, 1 prospective study	11–45 years	1995	2015	Weight gain and restoration, Estrogen replacement therapy, recombinant h-GH, recombinant h-IgF1, DHEA, Biphosphonates, Teriparatide
Robinson, L. et al. [50]	2017	Systematic review	MEDLINE; PsychINFO; Embase; Cochrane Database	19 studies =10 double-blind RCTs, 2 prospective observational studies, 1 retrospective cohort study, 1 case-control study and 5 non-randomised control trials	All	No limitation	3 March 2017	DHEA, various OC (EE or EE/levonorgestrel or EE/progestin or EE/Norgestimate), various oestrogen replacement treatments (transdermal 17ßPE/progesterone or oral EE/progesterone), Teriparatide (TPt), Alendronate, rhIgF1, Menatetrenone (MED) (vitamin K2), risedronate, transdermal testosterone
Brockmeyer, T. et al. [51]	2018	Systematic review	PubMed; Scopus; Web of Science	6 RCTs on medication (including one unpublished study)	All	October 2011 (post Watson 2012)	31 December 2016	Antipsychotics; Dronabinol; Tube feeding
Hale, M.D. et al. [52]	2018	Systematic review	PubMed; PsycINFO; CINAHL; Web of Science; Cochrane Library; Dissertations and Theses (ProQuest); Google Scholar	19 open, prospective RCTs, non-randomized controlled trials, prospective cohort studies, retrospective chart reviews	All	No limitation	September 2017	Tube feeding
Rizzo, S.M. et al. [53]	2018	Systematic review	PubMed; Scopus;Web of Science; PsycINFO	10 studies = 1 RCT, 1 prospective cohort study, 8 retrospective cohort studies	10–57 years	No limitation	May 2018	Enteral Nutrition via Nasogastric Tube

ED: Eating Disorder; BMI: Body Mass Index; EE: Ethinyl Estradiol; GH: Growth Hormone; OC: Oral Contraceptive; DHEA: Dehydroepiandrosterone; RCT(s): randomized controlled trial(s).

**Table 4:** Description of neuroleptics and antipsychotics studies [45,48].

Author	Study	Treatment Group	Daily Medication Dose	Length of Treatment	N	Mean Age ± SD (Years)	Results
Vandereycken and Pierloot, [77] 1982	Double-blind placebo controlled crossover	pimozide placebo	4 to 6 mg	6 weeks	18	Non reported	Non-significant on weight gain
Vandereycken, [78] 1984	Double-blind placebo controlled crossover	sulpiride /placebo sequence placebo /sulpiride sequence	300 or 400 mg	2–3 weeks	99	23.2 ± 6.5 23.7 ± 9.6	Non-significant on weight gain
Ruggiero et al., [65] 2001	Open-label	clomipramine fluoxetine amisulpride	Mean= 57.7 ± 25.8 mg Mean = 26.0 ± 10.3 mg Mean= 50.0 ± 0.0 mg	3 months	10 13 12	23.7 ± 4.6 4.5 ± 5.1 24.3 ± 5.8	No significant difference between groups in term of weight gain significant increase for fluoxetine and amisulpride groups
Cassano et al., [79] 2003	Open label	haloperidol	Months 1–3 Mean = 1.2 ± 0.4 mg; Months 4–6 Mean = 1.1 ± 0.2 mg	6 months	13	22.8 ± 4.2	BMI increased significantly in chronic and treatment-resistant patients
Mondraty et al., [80] 2005	Double-blind placebo controlled	olanzapine chlorpromazine	10 mg 50 mg	Mean = 46 ± 31 days Mean = 53 ± 26 days	87 10	25.3 ± 7.42 5.3 ± 7.3	No significant difference in weight gain
Brambilla et al., [81] 2007a	Double-blind placebo controlled	olanzapine and cognitive behaviour therapy and nutritional rehabilitation placebo and cognitive behavioural therapy and nutritional rehabilitation	2.5 mg for 1 month; 5 mg for 2 months	3 months	10	23 ± 4.8	No difference for weight gain
Brambilla et al., [82] 2007b	Double-blind placebo controlled	olanzapine and cognitive behavioural therapy placebo and cognitive behavioural therapy	2.5 mg for 1 month; 5 mg for 2 months	3 months	15 15	23.7 ± 4.8 26.3 ± 8.5	No difference for weight gain between groups greater improvement on the Eating Disorder Inventory ineffectiveness and maturity fear scores in the olanzapine group
Bissada et al., [83] 2008	Double-blind placebo controlled	olanzapine placebo	Start = 2.5 mg; Max = 10 mg – flexible dose regimen	10 weeks	16 18	23.6 ± 6.5 29.7 ± 11.6	Olanzapine: greater weight increase and faster achievement of weight goals
Attia et al., [84] 2011	Double-blind placebo controlled	olanzapine placebo	Start = 2.5 mg; Last 4 Weeks = 10 mg	8 weeks	11 12	27.7 ± 9.1	Olanzapine was associated with a small but significant increase in BMI compared to placebo
Kafantaris et al., [85] 2011	Double-blind placebo controlled	olanzapine and psychotherapy placebo and psychotherapy	Start = 2.5 mg; week 4 target = 10 mg	10 weeks	10 10	16.4 ± 2.2 18.1 ± 2.0	No significant difference in weight gain between groups
Hagman et al., [86] 2011	Double-blind placebo controlled	risperidone placebo	Mean = 2.5 ± 1.2 mg Mean = 3.0 ± 1.0 mg	17 weeks	182 2	16.2 ± 2.5 15.8 ± 2.3	No significant difference in weight gain between groups; the risperidone group showed greater reduction in drive for thinness over the first half of the study, but this was not sustained
Powers et al., [87] 2012	Double-blind placebo controlled	quetiapine placebo	Mean = 177.7 ± 90.8 mg	8 weeks	46	34 ± 14.5	No difference between quetiapine and placebo on weight, eating disorders, anxiety and depressive symptoms

BMI: Body Mass Index.

## Supplementary Materials

The following are available online at https://www.mdpi.com/2077-0383/8/2/278/s1, Table S1: Selected studies concerning innovative hormonal medications for female patients with AN and/or hypothalamic amenorrhea.
