# Supplementary material for: Medication in AN: A Multidisciplinary Overview of Meta-Analyses and Systematic Reviews
Source: J Clin Med. 2019 Feb 25;8(2):278. doi: 10.3390/jcm8020278 (PMC6406645; doi:10.3390/jcm8020278)
Supplement: Supplementary file 1 [file jcm-08-00278-s001.pdf]

Table S1: Selected studies concerning innovative hormonal medications for female patients with AN and/or hypothalamic amenorrhea.

**Author**	**Year**	**Patient**	**Age (Years Range) Sex**	**Pharmacological Agent**	**Type of Study**	**Follow-Up**	**Outcome Measure**
Welt C & al [1]	2004	HA IE+/LW+: 8<br>HA control: 6	19–33<br>F	Open-label Recombinant methionyl human leptin (r-metHu- Leptin): 0.08 mg per kilogram per day subcutaneously for two to three months, with 40% of the daily dose given at 8 a.m. and 60% at 8 p.m; third month: 0.2 mg per kilogram per day	Prospective study	4 weeks	Body composition<br>BMD<br>Pelvic ultra-sonography<br>Hormones (Leptin, FSH, LH, Inhibin, FreeT3, T4, TSH, Cortisol, ACTH, IgF1, IgFBP3)<br>Bone markers
Fazeli P.K & al [2]	2010	AN: 21<br>AN: rhGH+:10,<br>AN-rhGH-: 11	18–45<br>F	Recombinant human Growth Hormone (rhGH): mean max. daily SC injection dose: 1.4 ± 0.12 mg/d	Double blind RCT	12 weeks	IgF1, N-terminal propeptide of type 1 procollagen, type 1 collagen C-telopeptide, glucose, insuline, leptine, free fatty

							acids
Misra M & al [3]	2013	AN: 72<br>AN-E+: 38<br>AN-E-: 34	13–18<br>F	Transdermal 17 ßEstradiol (100 µg twice/week/2.5 mg of medroxyprogesterone acetate J1-J10/month)	Double blind RCT	18 mois	STAIC, EDI II, BSQ-34

Germain N & al [4]	2017	AN: 19<br>PHA: 15<br>SHA: 7	AN: 27.8<br>PHA:29.3<br>SHA:25.3<br>F	Gonadotropin-releasing hormone pulsatile therapy (portable SC infusion pump); 20 µg/90 minutes/4 weeks (induction cycle repeated if no pregnancy)	Retrospective observational monocentric study	Estradiol, LH, FSH, Progesterone, follicular recruitment (ultrasound), ovulation rate, pregnancy (living baby)
Leger J & al [5]	2017	AN: 10<br>AN-rhGH+: 10	13.3 ± 1.1 (civil age)<br>10.9 ± 1.7 (bone age)<br>F	Open-label Recombinant human Growth Hormone (rhGH): mean initial daily SC injection dose: 0.040 ± 0.006 mg/kg/d (mean duration of treatment: 3.6 ± 1.4 years)	Retrospective observational monocentric study	Height, HV, weight, pubertal status, serum IgF-I, glucose and glycated hemoglobin: at baseline and at 6-months interval

AN: Anorexia Nervosa; HA: Hypothalamic Amenorrhea; IE+: Incresead Exercice,;LW+: Low Weight; r-metHu- Leptin: recombinant methionyl human leptin; RCT: Randomized Controlled Trial,; PHA: Primary Hypothalamic Amenorrhea; SHA: Secondary Hypothalamic Amenorrhea; HV: Height Velocity; Rec-AN: weight recovered AN; STAIC: Spielberger's State-Trait Anxiety Inventory for Children; EDI II: Eating Disorder Inventory; BSQ-34 : Body Shape Questionnaire

References

1. Welt, C.K.; Smith, P.; Mantzoros, C.S. Recombinant Human Leptin in Women with Hypothalamic Amenorrhea. *N. Engl. J. Med.* **2004**, 11.
2. Fazeli, P.K.; Lawson, E.A.; Prabhakaran, R.; Miller, K.K.; Donoho, D.A.; Clemmons, D.R.; Herzog, D.B.; Misra, M.; Klibanski, A. Effects of Recombinant Human Growth Hormone in Anorexia Nervosa: A Randomized, Placebo-Controlled Study. *J. Clin. Endocrinol. Metab.* **2010**, *95*, 4889–4897.
3. Misra, M.; Katzman, D.K.; Estella, N.M.; Eddy, K.T.; Weigel, T.; Goldstein, M.A.; Miller, K.K.; Klibanski, A. Impact of Physiologic Estrogen Replacement on Anxiety Symptoms, Body Shape Perception, and Eating Attitudes in Adolescent Girls With Anorexia Nervosa: Data From a Randomized Controlled Trial. *J. Clin. Psychiatry* **2013**, *74*, e765–e771.
4. Germain, N.; Fauconnier, A.; Klein, J.-P.; Wargny, A.; Khalfallah, Y.; Papastathi-Boureau, C.; Estour, B.; Galusca, B. Pulsatile gonadotropin-releasing hormone therapy in persistent amenorrheic weight-recovered anorexia nervosa patients. *Fertil. Steril.* **2017**, *107*, 502–509.
5. Léger, J.; Fjellestad-Paulsen, A.; Bargiacchi, A.; Doyen, C.; Ecosse, E.; Carel, J.-C.; Le Heuzey, M.-F. Can growth hormone treatment improve growth in children with severe growth failure due to anorexia nervosa? A preliminary pilot study. *Endocr. Connect.* **2017**, *6*, 839–846.
